# Challenges and Design Strategies for Stable Zinc Anodes in Rechargeable Zinc Batteries

**DOI:** 10.1002/smll.202504170

**Published:** 2025-06-20

**Authors:** Hajra Khan, Chenyu Zhao, Karim Khan, Ayesha Khan Tareen, Asif Shahzad, Steven J. Langford, Hao Liu, Asif Mahmood, Guoxiu Wang

**Affiliations:** ^1^ Centre for Clean Energy Technology School of Mathematical and Physical Sciences Faculty of Science University of Technology Sydney Ultimo NSW 2007 Australia

**Keywords:** corrosion, dendrites, hydrogen evolution reaction, interface modification, structural engineering, Zinc ion battery

## Abstract

Zinc‐ion batteries (ZIBs) are increasingly recognized as promising candidates for large‐scale energy storage due to their high energy density, safety, low cost, and the natural abundance of zinc. However, the widespread adoption of ZIBs is limited by fundamental issues associated with the zinc metal anode, including dendrite formation, hydrogen evolution reaction (HER), passivation, self‐corrosion, and poor cycling stability. In recent years, substantial efforts have been made to address these challenges through approaches such as 3D current collector design, alloying, surface modification, and electrolyte engineering. This review provides a systematic, Zn‐anode‐focused summary of these advances, with emphasis on structural engineering, interface stabilization, and electrolyte tailoring to improve Zn^2^⁺ deposition behavior. Uniquely, this work integrates recent progress in advanced characterization techniques such as in situ/operando imaging and spectroscopy, to provide deeper insights into the failure mechanism of Zn anode materials. These details are critical in real‐time probing of interfacial and morphological evolutions upon charge/discharge. Finally, the review outlines the key future research directions are proposed to support the development of durable and high‐performance Zn‐based energy storage systems.

## Graphical abstract

Zinc metal anodes offer a promising pathway toward safer, low‐cost, and sustainable rechargeable batteries. This review presents a comprehensive overview of critical challenges—including dendrite formation, hydrogen evolution, and corrosion—and highlights recent advances in structural design, interface engineering, and electrolyte innovation. Strategies are discussed with a focus on practical relevance and future research directions.

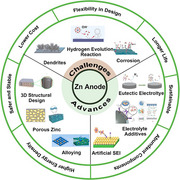

## 1 Introduction

Renewable energy is becoming a preferred source owing to an escalating demand for clean and green energy. Multiple strategies have been developed to produce efficient and sustainable energy, consisting of biomass energy, wind energy, thermal energy storage, geothermal energy, and solar energy.^[^ ^1^, ^2^, ^3^, ^4^ ^]^ However, such energy sources are remarkably challenging due to their intermittent nature, inconsistency, and transportation issues, which make them less favorable to meet modern‐day energy consumption.^[^ ^5^ ^]^ Alternatively, energy storage devices could provide a more competitive energy source due to their simplicity, safety, cleanliness, and ease of transport. Among diverse electrical energy storage ranges, batteries are more reliable because they are energy efficient, portable, and minimize greenhouse gas emissions.^[^ ^6^ ^]^ A prominent example is the lithium‐ion battery (LIBs), being commercially used since 1991 due to its high energy density, low self‐discharge rate, long calendar and cycle life, high efficiency, suitable for movable electronics and electrical automobiles.^[^ ^7^ ^]^ Despite exceptional characteristics, LIBs face several issues due to the highly reactive nature of Li, its higher cost, relatively low abundance, and combustible organic electrolytes.^[^ ^8^ ^]^ In addition, thermal runaway in LIBs is a major concern that can lead to overheating, internal short circuits, fire, and even explosions.^[^ ^9^, ^10^ ^]^ Therefore, it is imperative to develop alternative energy storage technologies that utilize abundant elements, are low‐cost, and are environmentally sustainable. Monovalent metal ions (e.g., Na^+^, K^+,^ etc.) have been thoroughly investigated in this regard but still face safety issues due to the highly reactive nature of these alkali metals and combustible organic electrolytes, which necessitate the search for safer alternatives.

**Figure 1:**
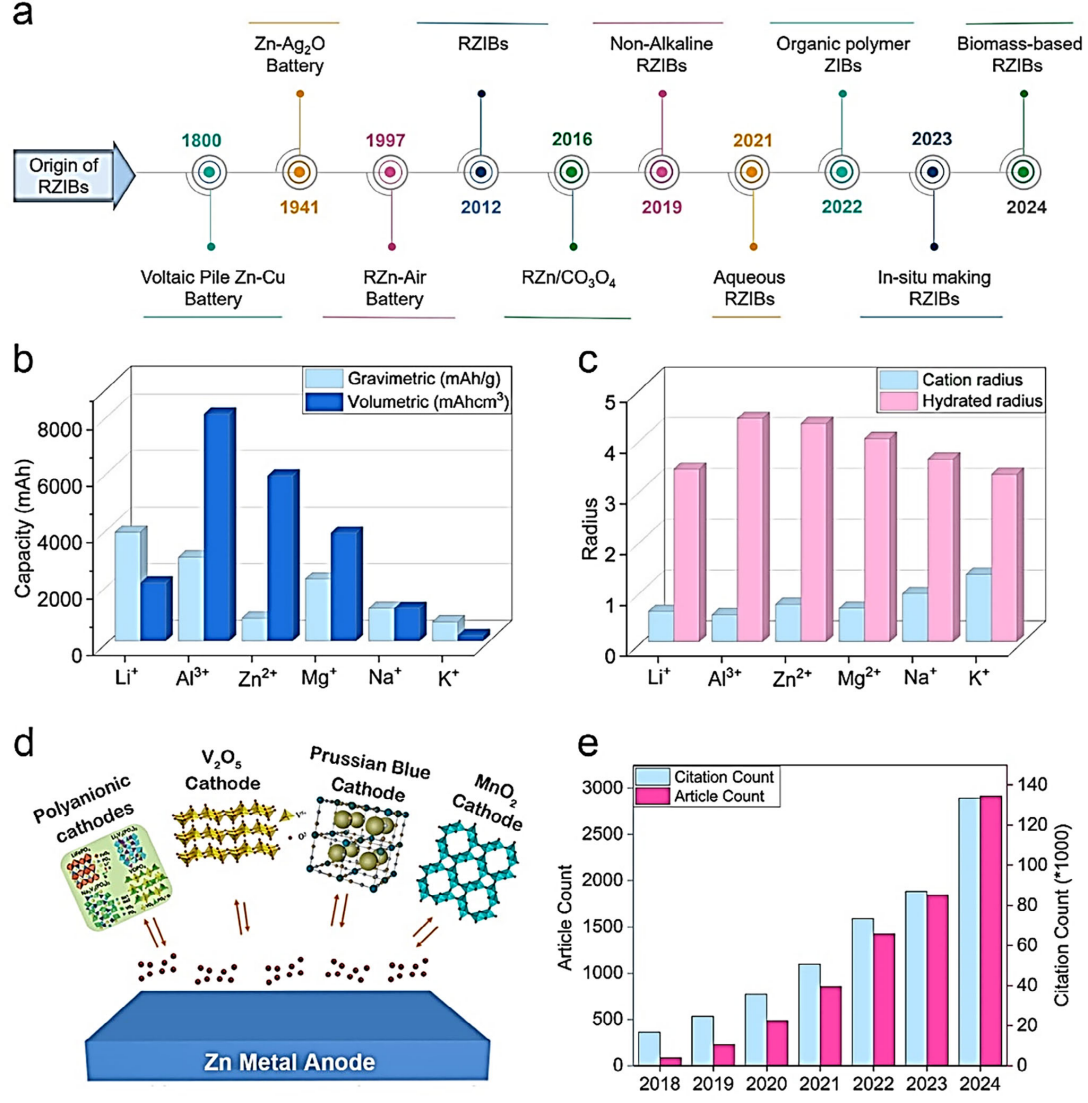
a) A timeline of the Zn‐based battery history from the beginning, b) a comparison of gravimetric capacity and volumetric capacity for the anode, and c) a comparison of cation radius and hydrated radius. d) variety of cathodes with Zn metal anodes. e) Progressive analysis of articles published in the past 7 years on ZIBs.

Batteries powered by multivalent metal ions (e.g., Ca^2+^, Mg^2+^, Zn^2+^, Al^3+^,) have also shown great promise and suitability for large‐scale energy storage systems (ESS), especially because these batteries can be enabled by aqueous electrolytes, which make them extremely safe.^[^ ^11^ ^]^ Rechargeable zinc ion batteries (RZIBs) perform via reversible insertion and extraction of Zn‐ion between anode and cathode during charge/discharge cycles. Among these diverse battery chemistries, Zn‐based batteries (e.g., Zn‐Cu, Zn‐Ag_2_O Zn‐air battery, Zn‐CO_3_O_4_, and non‐alkaline) have been intensively investigated because of the low reduction potential of Zn, which makes it ideal to use with an aqueous electrolyte system (**Figure** **1**a).^[^ ^12^, ^13^, ^14^, ^15^, ^16^, ^17^ ^]^ These Zinc ion batteries (ZIBs) utilize Zn as both an anode and as a charge carrier, intimating that Zn needs to be abundant and cost‐effective to make these batteries viable. Zinc is the 23^rd^ most abundant element on Earth's crust and the 4^th^ majorly extracted metal used in alloys, steel, pharmaceuticals, and chemicals.^[^ ^18^ ^]^ Zn emerged as a promising candidate as an anode material in Zn‐based batteries with high theoretical capacity (820 mAh g^−1^), low redox potential −0.76 V versus standard hydrogen electrode (SHE), and high volumetric capacity (5855 mAh cm^−3^), which enhance its benefit in volume‐restricted applications (Figure 1b).^[^ ^19^, ^20^ ^]^ Zinc has low electrical resistivity (5.91 µΩ cm) and a reduced ion size of 0.74 Å, facilitating faster ion transportation in the electrolyte.^[^ ^21^ ^]^ Moreover, Zn has a small hydrated radius (Figure 1c), which boosts fast ion diffusion, provides stable electrochemical kinetics, increases ionic conductivity, and supports ion intercalation for layer‐structured cathode material.^[^ ^22^ ^]^

Zinc metal serves as a highly enabling anode that can be integrated with a wide range of cathode materials, including Prussian blue analogs, MnO₂, V₂O₅, organic compounds, and polyanionic frameworks (Figure 1d).^[^ ^23^, ^24^, ^25^, ^26^, ^27^ ^]^ This broad compatibility highlights the versatility of Zn metal anode batteries across multiple ZIB chemistries. However, the large‐scale application of ZIBs suffer from the instabiligy of the Zn anode originating from dendrite formation, hydrogen evolution reaction (HER), surface passivation, self‐corrosion etc. These issues significantly hinder long‐term performance and limit the practical cycling life of the resulting ZIBs. Furthermore, the adaptability of ZIBs to a wide range of electrolyte systems (e.g., aqueous, organic, and gel etc.) add further complexity to developing universally effective stabilization strategies.^[^ ^28^, ^29^ ^]^ Concurrently, the push for temperature‐resilient energy storage has led to the design of Zn‐based batteries capable of operating under extreme thermal conditions, including sub‐zero temperatures (−30 to −40 °C) and elevated temperatures up to 70–80 °C, further intensifying the need for robust and scalable Zn anode designs. There is expeditious research toward stabilizing Zn anode using a range of strategies that focus on structure stabilization, interface engineering, as well as electrolyte design etc. The rapid increase in publications in this area reflects the growing urgency and scientific interest in addressing these limitations (Figure 1e). Furthermore, there is an evolving trend of utilizing high‐end and in situ characterization tools to understand the failure mechanism of the Zn anodes. For example, Zhao et al., worked on operando scanning electron microscopy to find the evolution in imaging the solid electrolyte interface.^[^ ^30^ ^]^ Therefore, there is a need to summarize this progress in an integrated form where all recent progress is deeply reviewed.

**Figure 2:**
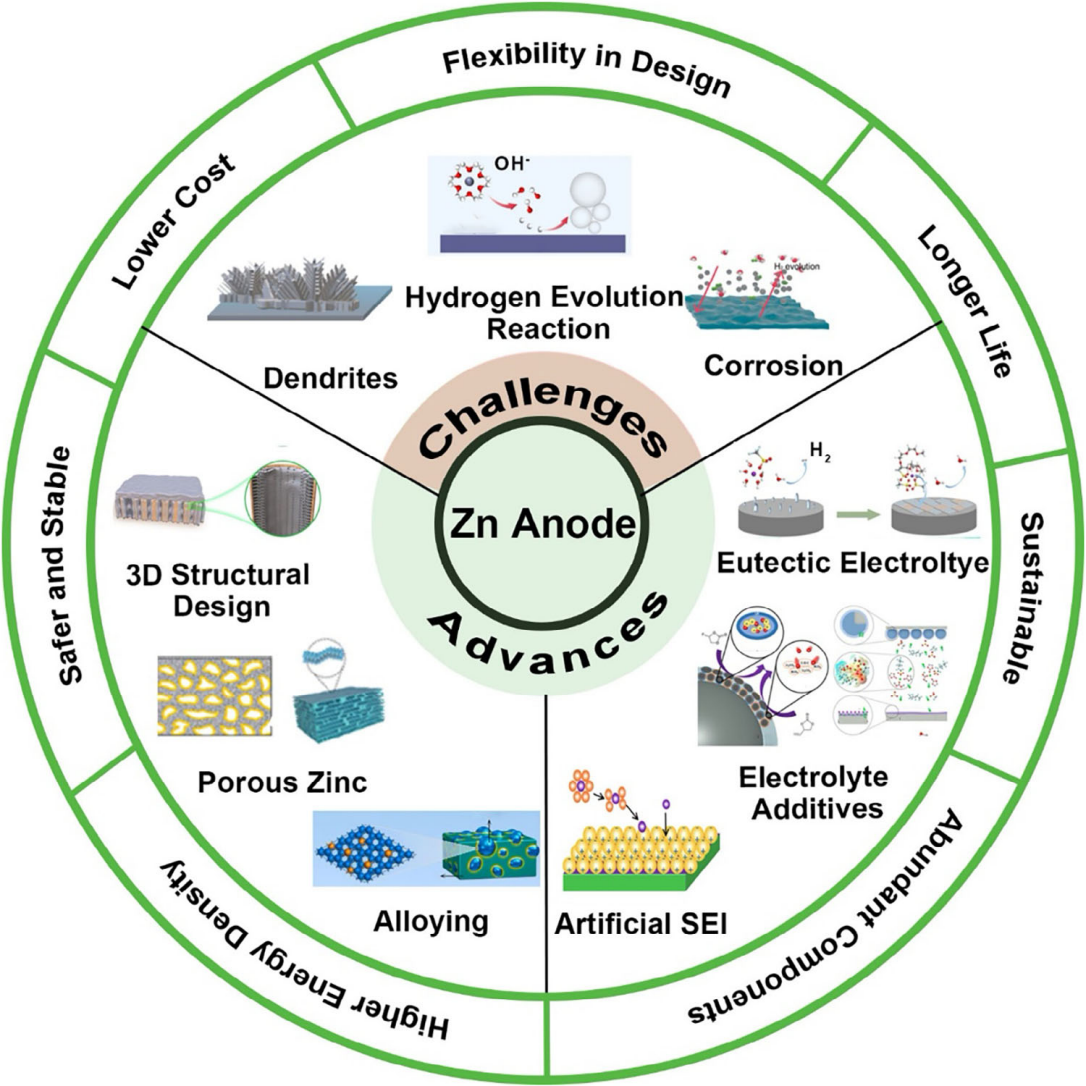
Overview of challenges and stabilization strategies for ZIBs.

Here, a systematic review is presented to discuss the challenges faced by Zn anodes thoroughly and put forward the recent progress to address these challenges to achieve high‐performing ZIBs. The review will first provide an overview of the challenges with Zn anodes such as dendrite growth, hydrogen evolution reaction (HER), self‐corrosion, and passivation which will be followed by a detailed discussion on recent strategies to overcome these challenges. It will particularly delve into structural engineering (e.g., 3D structural design, alloys or composite electrodes, etc.), interface engineering (e.g., artificial solid electrolyte interphase (ASEI), in situ‐SEI, and eutectic electrolytes) to stabilize the Zn anode, as summarized in **Figure** **2**.^[^ ^31^, ^32^ ^]^ In addition, this review will also provide deeper insights into the recent advancements in advanced characterization techniques, which have emerged as critical tools for visualizing interfacial evolution, identifying failure mechanisms, and correlating structural changes with electrochemical performance. These techniques are critical in providing key information about the dynamic behavior of Zn anodes under practical conditions, supporting the rational design of more robust systems. Finally, future research directions are proposed to guide the development of stable Zn anodes toward their full practical potential.

## 2 Challenges Faced by Zn Anodes

**Figure 3:**
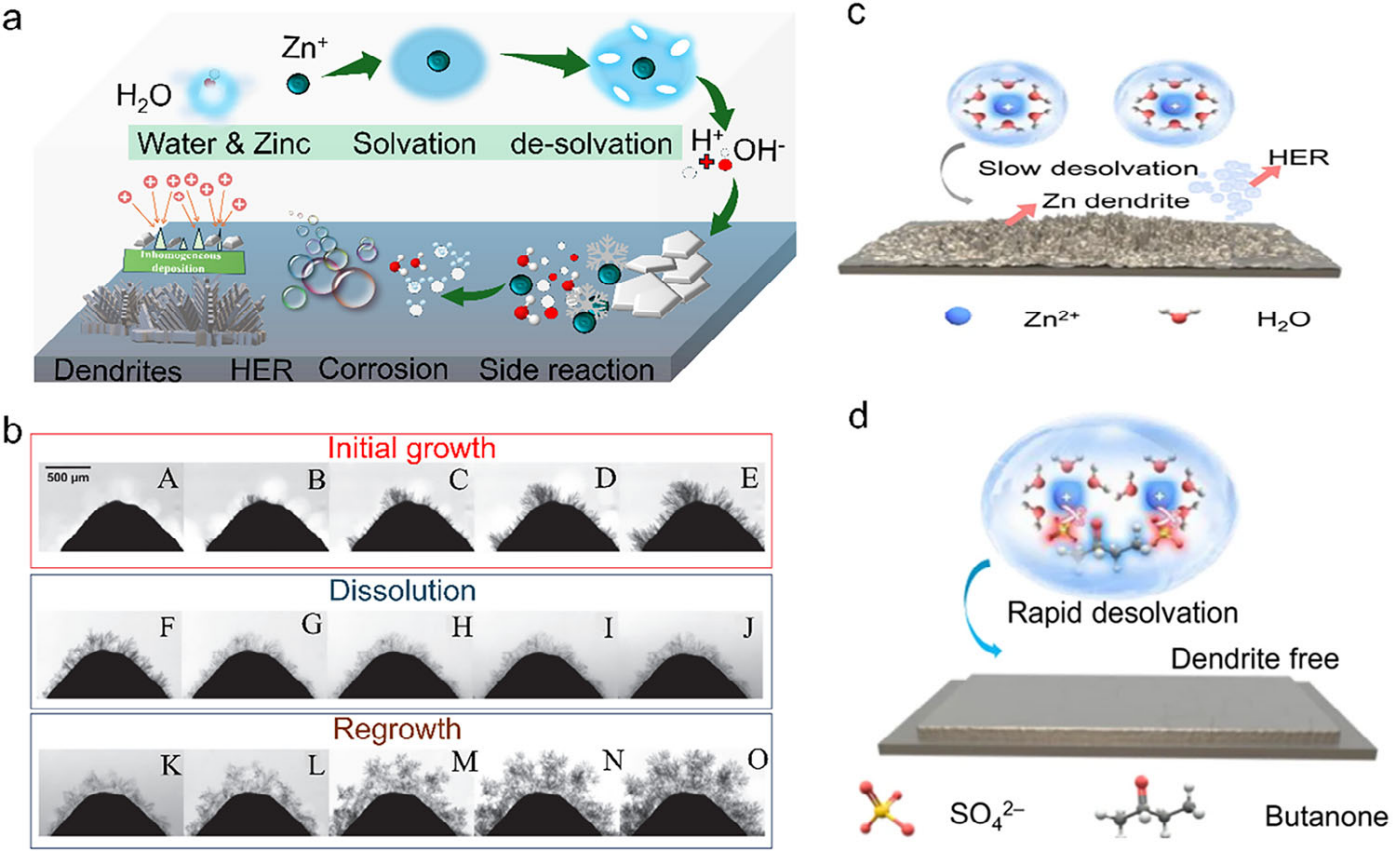
a) Diagram illustrating the solvation of Zn^2+^, interaction among electrode‐electrolyte surface, and series of side reactions, corrosion, HER, and dendrites. b) Operando study of Zinc dendrite growth, dissolution, and regrowth in the battery without separator at 30 mA cm^−2^. Zinc dendrite growth after (A) 214 s, (B) 388 s, (C) 561 s, (D) 734 s, and (E) 918 s. Zinc dendrite dissolution after (F) 10 s, (G) 229 s, (H) 447 s, (I) 655 s, and (J) 873 s. Zinc dendrite regrowth after (K) 122 s, (L) 589 s, (M) 1028 s, (N) 1485 s, and (O) 1953 s, Reproduced with permission.^[^ ^39^ ^]^ Copyright 2019, Elsevier. Schematic illustration of Zn anode with c) slow desolvation kinetics on Zn plating and d) Rapid desolvation enabling uniform Zn plating, Reproduced with permission.^[^ ^42^ ^]^ Copyright 2024, Springer.

The Zn anode faces challenges that obstruct its performance and commercial potential, such i) dendrite formation promoted by uneven deposition of Zn^2+^ leading to needle‐like pointed structures, that leads to short circuits, and where side reactions cause degraded battery capacity over time ii) HER reaction on the Zn surface, iii) surface passivation layer which imparts sluggish charge transfer kinetics, and iv) corrosion as summarized in **Figure** **3**a.^[^ ^33^, ^34^, ^35^ ^]^ The coming section will provide a detailed discussion of these challenges and strategies.

### 2.1 Zinc Electrodeposition and Dendrite Growth

The Zn ions are reversibly stripped and plated on the electrode surface during the charge and discharge process. Ideally, this process should be uniform, resulting in smooth Zn plating in successive cycling. However, in an actual battery operation, the surface irregularities and high‐energy sites provide preferential sites for Zn deposition. Due to the preferential nucleation of Zn ions on such sites, small Zn islands are formed on the electrode surface, which grow with successive cycling in the form of needle‐like structures called dendrites. These dendrites have the potential to pierce the separators and short‐circuit the battery, eventually leading to battery failure. It is important to understand the nature of dendrites and the growth factors in different types of Zn battery systems to propose methods to limit their growth. The Zn anode is unique because of its compatibility with aqueous electrolytes, which brings forward various challenges, especially the Zn stripping/plating in electrolytes over diverse pH ranges. The Zn deposition mechanism varies depending on the electrolyte's pH; therefore, the factors governing the dendrite growth also vary depending on the pH of the electrolyte. It is widely reported that the Zn dendrites are more pronounced in alkaline solutions because zinc exhibits a higher electrochemical activity in alkaline electrolytes, which is thermodynamically unstable. In alkaline electrolytes, Zn first combines with hydroxide and water to form zincate ions (Zn(OH)_4_^2−^) followed by reduction into metallic zinc by obtaining electrons on the Zn surface in the charging process, as shown in Equation (1).

(1) $$\mathrm{ZnO}+2OH^{-}+H_{2}O\to \mathrm{Zn}{\left(\mathrm{OH}\right)}_{4}^{2-\;}$$

(2) $$\mathrm{Zn}{\left(\mathrm{OH}\right)}_{4}^{2-\;}+2e^{-}\to \mathrm{Zn}+4OH^{-\;}$$

Similarly, the electrochemical deposition of zinc occurs directly on the Zn surface without the formation of zinc oxide and zincate ions in neutral electrolyte. The direct reduction of Zn ions on the Zn metal anode is attributed to insufficient concentration of OH^−^ ions in the neutral electrolyte, which limits the formation of zinc oxide and zincate ions in Equations (2) and (3).^[^ ^36^, ^37^ ^]^ Regardless of the deposition mechanism, the formation of dendrites is still observed on the Zn surface in all electrolyte systems. Apart from electrolyte pH, zinc dendrite formation is further dependent on local electrolyte concentration and solvation chemistry of the metal ions in the electrolyte solutions.^[^ ^38^ ^]^

(3) $$\mathrm{Zn}⇆Zn^{2+}+2e^{-}$$

Dendrite growth in ZIBs can be described as the following stages: initial growth, dissolution, and regrowth. Yufit et al. introduced an Operando study for dendrite growth to understand the phenomenon in depth.^[^ ^39^ ^]^ It was observed that the dendrites did not start growing until a certain overpotential was reached, where the time taken during the initial growth of dendrites could be ascribed to several factors such as concentration, local current density, and temperature. A needle‐like structure was detected during the early charging stages onto a cone‐shaped Zn metal anode in the presence of aqueous zincate electrolyte after 214 s of deposition (Figure 3b(A)). After 388 and 561 s of deposition (Figure 3b(B,C)), respectively, dendrites start growing at several other locations on the cone electrode. Interestingly, HER was also observed owing to an increasing growth of dendrites, which can be seen as white semicircles in the initial growth. Continuous Zn‐electrodeposition caused the growth of secondary dendrites, which are visible and prevail after 734 and 918 s (Figure 3b(D,E)), respectively. These secondary dendrites grow on the body of primary dendrites instead of growing directly on the electrode tip, and ternary dendrites also come out from the body of secondary dendrites. The subsequent application of 10 mA current for 15 min leads to electro‐dissolution or the narrowing of needles and branches‐like structures (Figure 3b(F,G)). This narrowing starts from the upper side of the dendrite and penetrates down to the electrode tip until it is no longer connected to the tip and dissolves itself (Figure 3b(H–J)). Upon reversing the polarity, regrowth of the dendrites was observed, along with detached dendrites spontaneously moving upward (Figure 3b(K–O)). The solvation shell formed around the Zn^2+^ in the electrolyte plays a strong role in the homogeneous stripping‐plating of Zn on the electrode surface.^[^ ^40^ ^]^ Generally, a weak solvation shell minimizes the growth of dendrites and side reactions in ZIBs. The significant factor that estimates the solvating capability of any solvent are the donor number (DN) and dielectric constant (ε), where (ε) represents electrostatic force among DN and solvated ions. So, the solvating power of water could be limited by additives or some other strategies with low (ε) and DN.^[^ ^41^ ^]^ Shi et al., explained the elimination of solvated water and enhancing the desolvation kinetics of Zn^2+^ to inhibit the dendrite growth and other side reactions on the Zn anode. During the desolvation process of [Zn(H_2_O)_6_]^2+^, a huge number of water molecules are set free and come into contact with the Zn anode, affecting the battery performance due to increased polarization, impeding ion migration, initiation of dendrite growth, and also impacting SEI formation which activated HER and corrosion (Figure 3c).^[^ ^42^ ^]^ Meanwhile, during the rapid desolvation process, the nucleation overpotential was less at a high current density (1‐10 mA cm^−2^), favoring the homogeneous growth of Zn deposits (Figure 3d).^[^ ^43^ ^]^ As a result, a combination of these factors leads to the formation of dendritic structures that enhance the surface area of the anode and further stimulate side reactions such as HER, corrosion, and passivation.

### 2.2 Hydrogen Evolution Reaction (HER)

HER constitutes another major challenge for Zn anode stability as it is unavoidable in neutral or mildly acidic electrolytes, leading to decreased capacity, increased impedance, and ultimately electrolyte leakage. HER is the result of insoluble by‐products and depends upon the concentration of H^+^.^[^ ^44^ ^]^ For instance, owing to the lower electronegativity of Zn to hydrogen, Zn atoms react with H_2_O present in the ZnSO_4_ electrolyte, which promotes HER, alters the electrolyte composition, and leads to the formation of a strong solvation shell, which sponsors dendrite formation.^[^ ^45^ ^]^ As a process, HER not only causes electrode corrosion but also leads to internal pressure in the battery, leading to battery damage and electrolyte leakage.^[^ ^46^ ^]^ The H_2_ generation in the ZIBs is generally governed by the following Equations ((4), (5), (6)).

(4) $$2H^{+}+2e^{-}\to H_{2\;}$$

(5) $$2H_{2}O+2e^{-}\to H_{2}+2OH^{-}$$

(6) $$\mathrm{Zn}+2H_{2}O\to \mathrm{Zn}{\left(\mathrm{OH}\right)}_{2}+H_{2}$$

**Figure 4:**
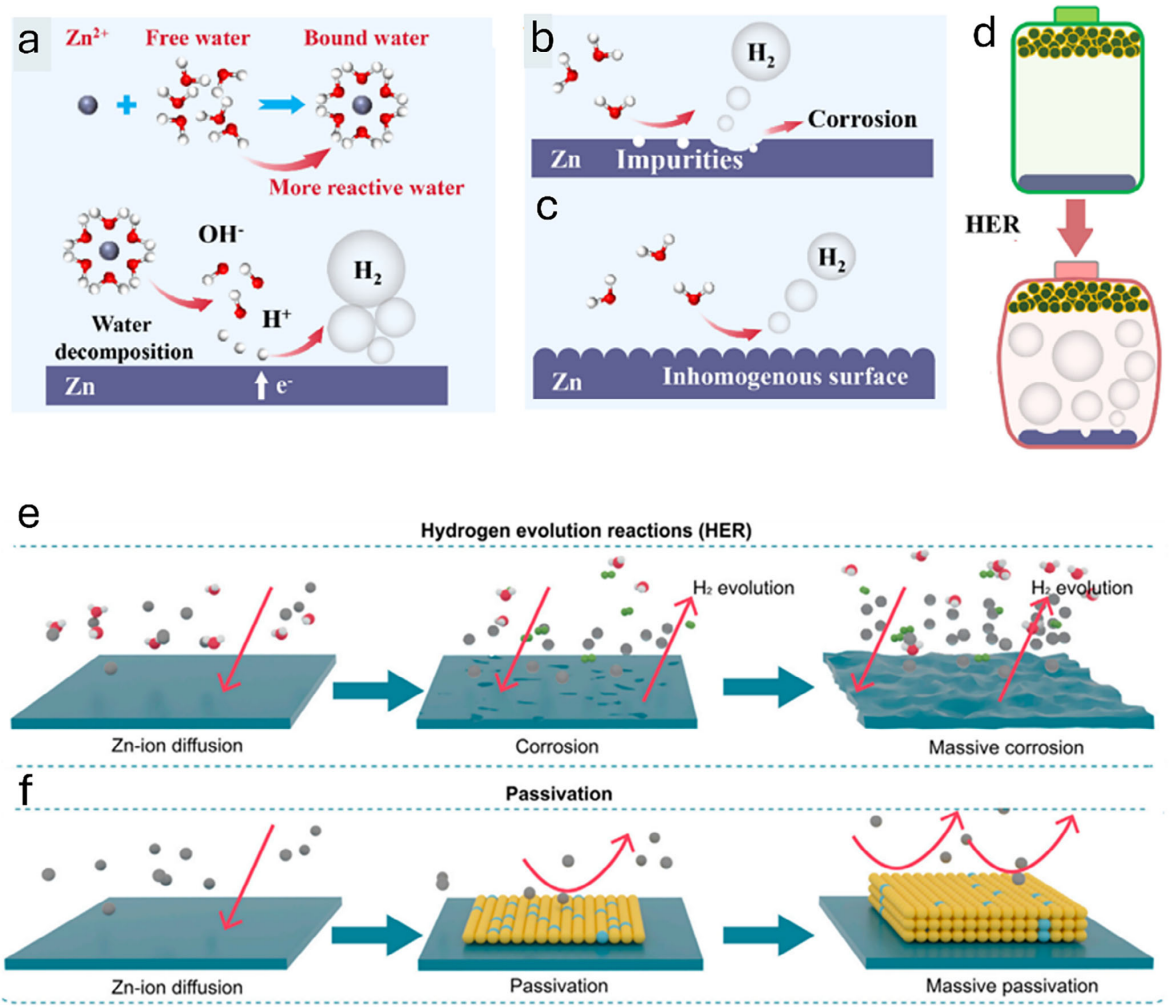
a–c) The HER mechanism for ZIBs in aqueous electrolytes, d) Deprotonation energies of free H_2_O and Zn (H_2_O)_6_^2+^, Reproduced with permission.^[^ ^48^ ^]^ Copyright 2023, Elsevier. e) free‐energy diagram for the HER, f) Scheme shows battery expansion caused by HER. Reproduced with permission.^[^ ^49^ ^]^ Copyright 2021, Elsevier.

HER on the Zn anode can initiate for several reasons (**Figure** **4**a–c). The first reason is the formation of the Zn(H_2_O)_6_^2+^ solvated structure by solvation of Zn^2+^ in the aqueous electrolyte. This solvated structure is robust, owing to the intense polarization in the ZIBs. Due to this strong interaction, electron transfer from Zn to water molecules to break the solvation shell leads to the weakening of the O‐H bond in water molecules. Thus, water molecules in Zn(H_2_O)_6_^2+^ are more reactive and facile to reduce than free water, leading to the production of H_2_ at the Zn electrode interface. Therefore, in an aqueous environment, the intermolecular H‐bond assists the movement of protons and OH^−^ through the Grotthuss diffusion mechanism, generating HER. In addition to charge transfer, the impurities in the anode, such as the presence of carbon and nickel, can promote H_2_ generation. The metallic impurities provide suitable binding energies for the preferential adsorption of water moieties from the electrolyte and decompose water to H_2_, leading to enhanced HER. The impact of impurities is much more pronounced in acidic electrolytes due to a surplus of H^+^ ions. It has been proposed that the impurities act as the cathode to make a micro‐cell in the Zn anode, causing HER and the corrosion of the Zn metal. For example, in ZnSO_4_ electrolyte, the electrochemical corrosion and HER in this micro‐cell can be stated as Equation (7).

(7) $$\mathrm{Zn}+2H^{+}\to H_{2}↑+Zn^{2+}$$

In addition, the surface atomic structure substantially affects the electrochemical characteristics of Zn anodes. Typically, the Zn electrode has an irregular surface morphology at the nanoscale because of the disordered crystal planes of Zn metal, which indicates heterogeneous electron and ion distribution on the Zn electrode surface. Specifically, the crystal planes of Zn with the hexagonal close‐packed (HCP) structure generally include Zn (002), Zn (100), and Zn (101), varying significantly for each crystal plane, specifically for the Zn (100) and Zn (002).^[^ ^47^ ^]^ The Zn (100) surface atoms are attached in an uneven, wavy pattern, leading to non‐uniform interface charge density and Zn^2+^ flux, which resist dendritic growth. On the contrary, the Zn (002) crystal surface is relatively flat and shows homogeneous interfacial charge density, which benefits the uniform deposition of Zn^2+^.

Moreover, the electrochemical activity of the Zn (002) surface reduces because the surface energy of Zn (002) is minimal, which is favorable for weakening the HER. Overall, HER appears on the surface of the Zn metal anode due to water decomposition via electrochemical processes. Consequently, HER will constantly consume fresh Zn electrodes and electrolytes, causing quick capacity decay and degradation of ZIBs. Moreover, the production of H_2_ increases the inner pressure of the assembled battery, which further leads to increased polarization, expansion, and even explosion, causing non‐negligible safety issues (Figure 4d). In this regard, several strategies are proposed to mitigate the production of hydrogen gas by modifying electrolytes and finding ways to reduce the pressure inside the battery, which are thoroughly discussed in the following sections.^[^ ^50^ ^]^

### 2.3 Passivation and Corrosion in the Zn Anode

Passivation in the Zn anode is another factor affecting ZIB performance, resulting from side reactions on the Zn surface. Two types of corrosion are generated during the cycling process in aqueous ZIBs: electrochemical corrosion and self‐corrosion. Electrochemical corrosion occurs in mildly acidic electrolytes because of the oxidation of the Zn‐metal anode. During the discharge, Zn^2+^ makes bonds with anions and produces by‐products.^[^ ^51^ ^]^ A redox reaction occurs inside the electrolyte and incorporates the intercalation of H^+^ and HER. Eventually, this phenomenon results in Zn hydrosulfates (ZHS) production, growing in particulate points where the proton has been consumed. In such a scenario, OH^−^ and sulfate ions (SO_4_^2−^) accumulate with Zn^2+^.^[^ ^52^ ^]^ These insoluble leftovers reduce the functional reaction of Zn anode surface space, inducing a sluggish Zn‐ion diffusion throughout the interphase. Hence, the surface area of the anode lowers, causing bigger battery polarization and promptly lowering the cyclic performance. The standard potential of H^+^ is always larger than Zn^2+^, particularly in aqueous mild‐acidic electrolytes. The proton (H^+^) gets ready to receive electrons from the metallic anode.^[^ ^53^ ^]^ Therefore, hydrogen gas is generated, causing Zn corrosion and transforming Zn metal into Zn^2+^. This phenomenon exerts internal pressure on the battery cell, hence initiating self‐corrosion. Liu et al. explained the detailed corrosion phenomenon through irregular Zn‐ion diffusion and HER through massive corrosion.^[^ ^49^ ^]^ Likewise, the corrosion and passivation of Zn anodes hold back the service of secondary ZIBs owing to the poor thermodynamic stability in the aqueous solution, and parasitic self‐discharge reaction may happen and produce H_2_ (Figure 4e), corroding the electrodes and lowering the columbic efficiency (CE). When Zn in the electrolyte goes beyond the saturation limit, a solid precipitate forms in the cell, like ZnO (in alkaline electrolyte) and Zn_4_(OH)_8_SO_4_ (in mildly acidic or neutral electrolytes), which may cause the passivation of the electrode (Figure 4f). The development of passivation films will deter the transport of chemical species among electrodes and electrolytes, consequently dropping the electrochemical performance and causing the abrupt death of batteries. This summarizes that the mitigation of dendrites and side reactions is necessary by opting for some stabilization strategies, as explained in the next section.

## 3 Stabilization Strategies for High‐Performance Zn Anodes

To overcome several challenges faced by Zn anode as summarized above, a range of different strategies have been proposed to increase their longevity and charge‐discharge efficiency. The major strategies employed in this regard include structural engineering of the Zn anode via artificial coating to protect the Zn anode interface, modifying the electrolyte chemistry using additives to improve the cycling of the Zn anode, etc. These strategies are thoroughly discussed in this section to give a deeper insight into the recent progress.

### 3.1 Structural Engineering of Zinc Anodes

Under applied conditions, pure zinc metal can pass through various phases, but a hexagonal close‐packed (HCP) structure with the (002) plane is more favorable for Zn plating, with the lowest surface energy of 0.02 eV Å^−1^. The geometrical characteristics of electrochemically deposited zinc predominantly depend on the zinc nucleation and growth procedures. These processes are theoretically operated by the zincophilicity of the substrates and the local current or electric field division covering the substrates. The direct exposure of Zn anodes to aqueous electrolytes poses a challenge, especially the growth of Zn dendrites and other side reactions, as well as corrosion.^[^ ^54^ ^]^ To address these challenges, modulation of the Zn anode structure is a promising approach.^[^ ^5^, ^55^ ^]^ The modulation, including face engineering, nanostructuring of materials, porosity of the Zn anode, and alloying, etc., can promote Zn nucleation and growth, induce Zn deposition, regulate Zn ion flux, and inhibit HER and corrosion.^[^ ^56^, ^57^, ^58^ ^]^ For zinc anode substrate adjustment, the recent developments in modification strategies, including 3D structural design and zincophilicity regulation, are summarized here. This provides clear insights toward directions for future research of stable zinc anodes and assists in expediting their practical applications in ZIBs.

#### 3.1.1 3D Structural Design for Homogenous Mass Transfer

Zn anodes with porous 3D structures are an effective strategy to enhance the performance of ZIBs. This is because the abundant porous structure increases the surface area of the Zn anode, which can provide more sites for Zn^2+^ and reduce the local current density, thus achieving uniform deposition of Zn^2+^ and rapid ion transfer, preventing the dendrite growth of Zn that enhances the cyclability.^[^ ^55^ ^]^ The electric and ionic concentration field encompassing the anode substrate performs a substantial function in metal deposits' nucleation and growth processes. The usual substrates of zinc metal anode are planar; the uniform electric field will be disturbed when a minor protrusion is formed on the planar structure. The reconstructed electric field concentrates near the protrusions, escorting to intensified deposition and eventually causing dendrite growth. 3D substrates with increased specific surface areas (SSA) can homogenize current density, efficiently lowering it below the critical current density for dendrite growth during charging and discharging. Subsequently, metal deposits favorably form inside the framework rather than on the outer surface of 3D substrates, thus preventing dendrite formation. Furthermore, 3D substrates with specifically controlled nanopores can encourage the formation of interface‐confined concentrated electrolytes. This efficiently protects zinc anodes because the unique electric double layer (EDL) reduces free H_2_O and rich zinc ions.^[^ ^59^ ^]^ 3D substrates could relieve the capacity loss of zinc anodes throughout calendar aging, compared to planar substrates, where 3D hosts with the same zinc loading exhibit an insignificant loss of Coulombic efficiency. This improvement is ascribed to the substantially bigger surface areas of 3D substrates, which can enable faster mass transfer and prevent the growth of inactive zinc.

**Figure 5:**
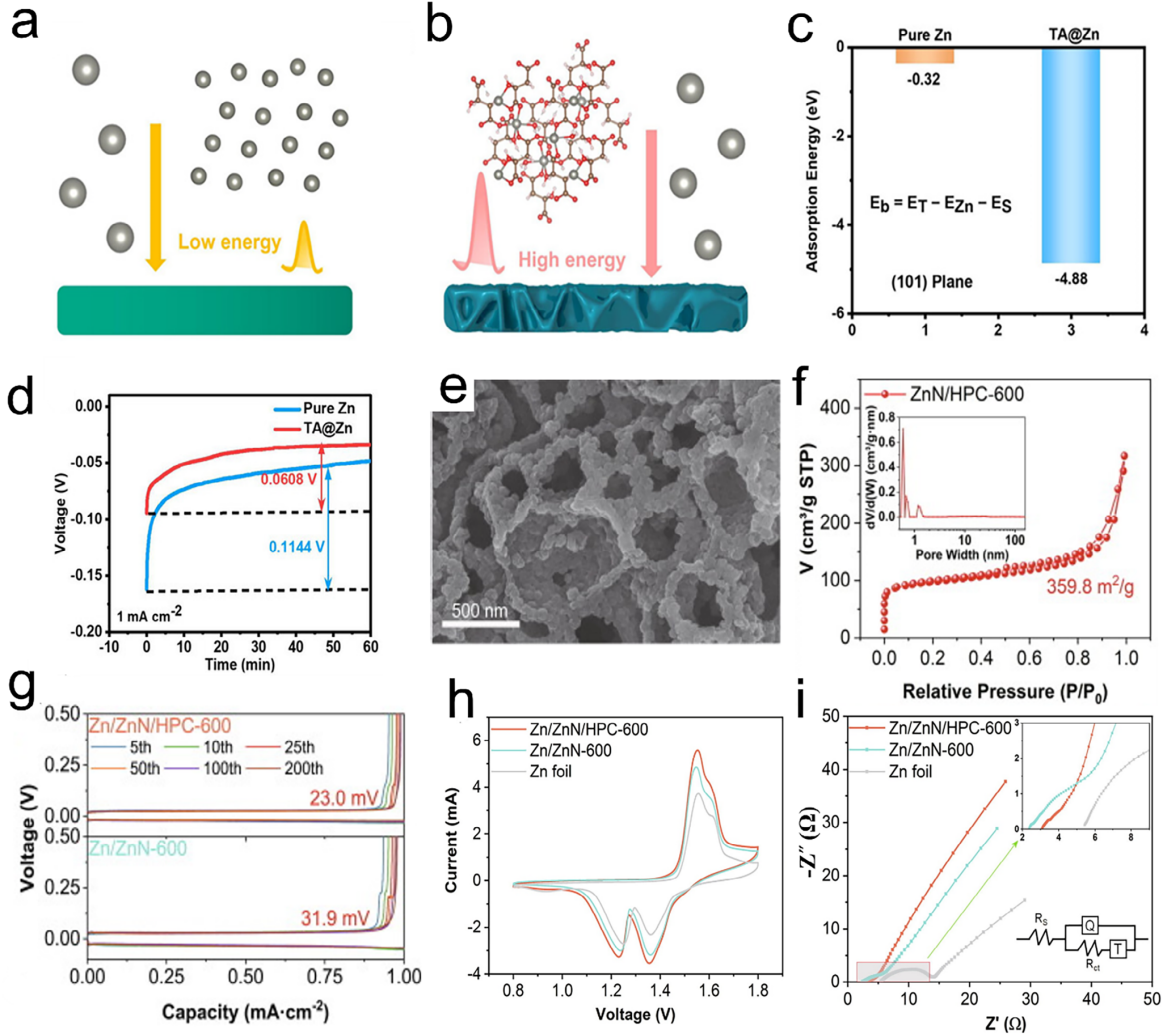
Graphical representation of the adsorption of a) Zn foil and b) TA@Zn. c) Calculating adsorption energy in the (101) plane for Zn foil and TA@Zn. d) Nucleation overpotential of Zn^2+^ on bare Zn and TA@Zn surfaces at 1 mA cm^−2^ current density, Reproduced with permission.^[^ ^60^ ^]^ Copyright 2023, Wiley‐VCH. e) SEM image of ZnN/HPC‐600. f) Nitrogen adsorption and desorption isotherms of ZnN/HPC‐600 with inset curves illustrating the pore size distribution. g) Charge‐discharge curves of Zn/ZnN‐600 and Zn/ZnN/HPC‐600 at a current density of 1.0 mA cm^−2^. h) CV curves of Zn, Zn/ZnN‐600, and Zn/ZnN/HPC‐600 at a scan rate of 0.1 mV/s. i) Nyquist plot of Zn, Zn/ZnN‐600, and Zn/ZnN/HPC‐600, Reproduced with permission.^[^ ^61^ ^]^ Copyright 2023, Elsevier.

Sun et al., designed a protective layer of zinc tartrate (C_4_H_4_O_6_Zn) by interfacial reaction between metallic Zn and tartaric acid as the anode of ZIBs, and the surface of Zn metal can be changed into a loose and porous structure, which is beneficial for the rapid diffusion of Zn^2+^ and the improvement of reaction efficiency.^[^ ^60^ ^]^ **Figures** **5**a–c demonstrates the DFT calculations of bare Zn and TA@Zn for Zn^2+^, and the adsorption energy on the TA@Zn surface (−4.88 eV) is much lower than that of metallic Zn (−0.32 eV), which indicates that the structure of TA@Zn is more favorable for the uptake of Zn^2+^. The nucleation potential was only 0.0608 V at a current density of 1 mA cm^−2^, which was much lower than that of 0.1144 V for bare Zn (Figure 5d) and lower than the nucleation site potential that could effectively promote the plating‐stripping of Zn^2+^ on the anode and mitigate the potentiodynamic polarization during the cycling process. Moreover, in the symmetric battery, the TA@Zn anode achieves a stable cyclability of 5000 h at a current density of 1 mA cm^−2^ and 130 h even at a high current density of 20 mA cm^−2^. Tang et al., prepared Zn‐nitrogen co‐doped 3D honeycomb porous carbon (HPC) by confined growth on the walls of HPC nano‐pools and obtained after pyrolysis in argon atmosphere to get the final product ZnN/HPC‐600 (Figure 5e) as an anode for ZIBs.^[^ ^61^ ^]^ As can be seen from the results of N_2_ isothermal adsorption and desorption tests (Figure 5f), the characteristic I(b) adsorption curve of ZnN/HPC‐600 proves that it has a microporous structure and the H4‐type hysteresis loop at relative pressures between 0.45 and 0.90 demonstrates that ZnN/HPC‐600 has a narrow slit mesoporous structure. The Zn/ZnN‐600, which has no porous structure, was introduced in the project for comparison during the tests. The test results of the charge/discharge curves for 200 cycles at a current density of 1.0 mA cm^−2^ (Figure 5g). This shows that after 200 cycles, the voltage lag (23 mV) of the porous structure Zn/ZnN/HPC‐600 in the symmetric cell remains unchanged and is smaller than that of Zn/ZnN‐600 (31.90 mV), confirming that the porous structure is beneficial to the cyclability of the anode.

CV tests were performed on Zn/ZnN/HPC‐600, Zn/ZnN‐600, and bare Zn anodes in a Zn‐ion full battery with a MnO_2_/HPC cathode (Figure 5h) for comparison. The two peaks at 1.37 and 1.61 V represent the intercalation and vapor extraction of H^+^, and the two peaks at 1.26 and 1.55 V represent the intercalation and vapor extraction of Zn^2+^. The peak current of the battery with Zn/ZnN/HPC‐600 anode is higher than that of the Zn/ZnN‐600 battery, suggesting that the pore structure contributes to the enhancement of Zn^2+^ loading. Moreover, the change in peak position can be attributed to the different affinity for Zn^2+^. The Zn/ZnN/HPC‐600 anode has a lower transfer resistance (2.58 Ω cm^2^) than the Zn/ZnN‐600 (12.58 Ω cm^2^) and Zn anodes (13.76 Ω cm^2^) (Figure 5i),. Thus, the HPC with porous holes also enhances the conductivity of the anode. In the end, the full battery with Zn/ZnN/HPC‐600 as the anode maintains 96% (CE) after 1000 cycles at 0.50 A g^−1^ and close to 100% CE at 5 A g^−1^ after 6000 cycles.

**Figure 6:**
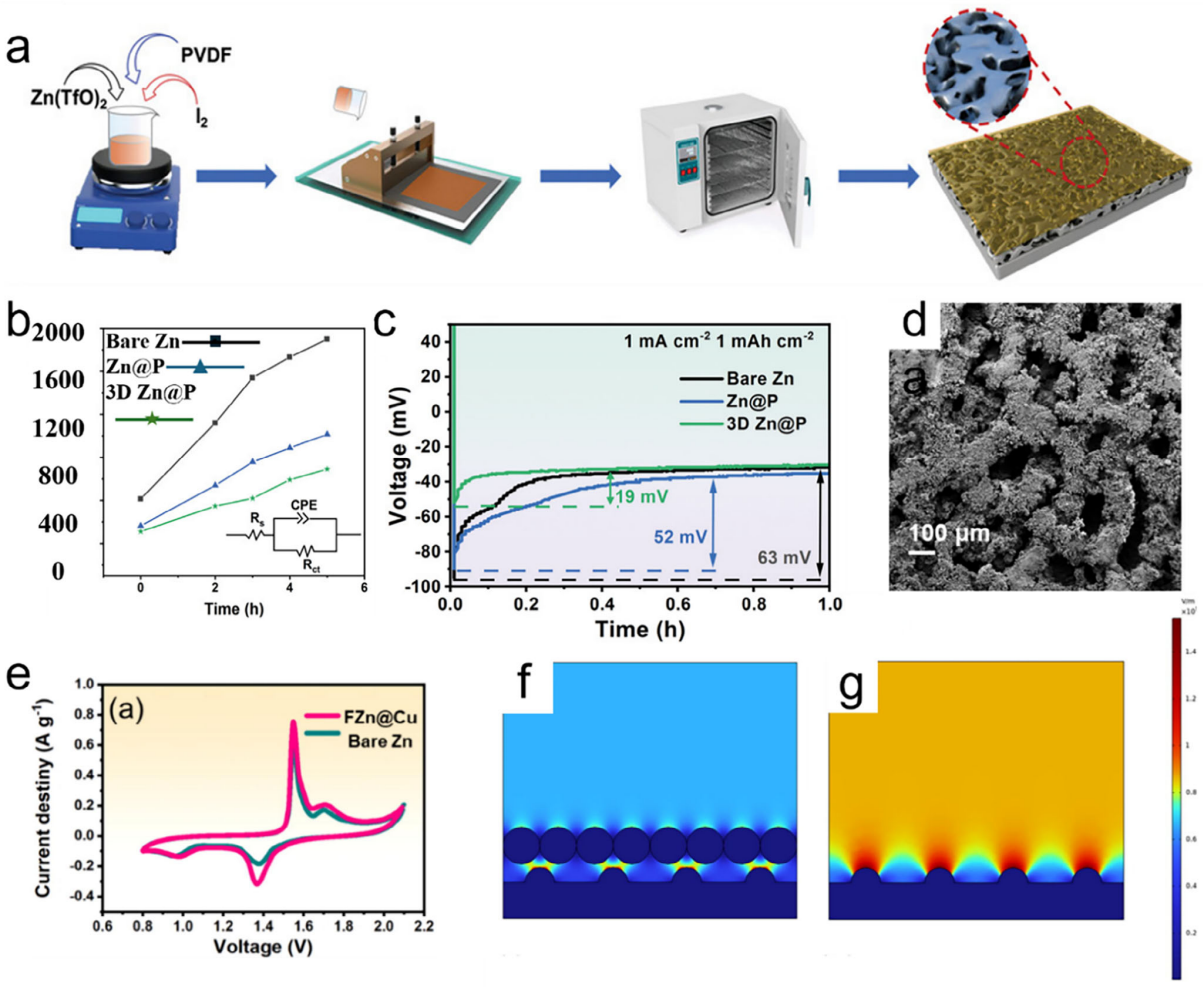
a) Schematic flow diagram for preparation of 3D Zn@P. b) R_ct_ results with time for different symmetric batteries (Zn, Zn@P, and 3D Zn@P). c) Voltage‐time curves during Zn nucleation on Zn, Zn@P, and 3D Zn@P anodes at 1 mA cm^−2^ current density. Reproduced with permission.^[^ ^63^ ^]^ Copyright 2023, Wiley. d) SEM images of FZn@Cu. e) CV curves of FZn@Cu and bare Zn anodes at a sweep rate of 0.2 mV s^−1^ in a full battery with MnO_2_ as a cathode. Simulated electric field profiles of f) FZn@Cu and g) pristine Zn. Reproduced with permission. Reproduced with permission.^[^ ^64^ ^]^ Copyright 2024, Elsevier.

Wang et al. prepared 3D porous Zn anodes with PVDF films (3D Zn@P) by mixing PVDF, Zn(TfO)_2,_ and I_2_ in N‐methyl pyrrolidone (NMP) solvent for etching of Zn foils by a simple squeegee casting method. Zn anodes with PVDF films (Zn@P) were also prepared for comparison (**Figure** **6**a).^[^ ^62^ ^]^ Comparison in the variation of Rct with time for bare Zn, Zn@P, and 3D Zn@P in symmetric batteries (Figure 6b). Where Zn@P has the smallest rise in impedance compared to Zn@P and bare Zn, especially the rise in impedance of 3D Zn@P for the sink battery (692.68 Ω) is much lower than that of the bare Zn symmetric battery (1899.87 Ω), which indicates that 3D Zn@P has fewer side reactions and corrosion in the sinking battery. Moreover, the decreasing slope of the R_ct_‐time curve proves that the modification further enhances corrosion resistance. The excellent corrosion resistance of the 3D Zn@P anode can be attributed to the PVDF film preventing the electrodes from direct contact with the electrolyte. The three‐position porous structure can regulate the concentration gradient of Zn^2+^. Moreover, the voltage‐time curve (Figure 6c) shows that the Zn@P nucleation overpotential of 3D Zn@P is significantly lower (19 mV) than that of Zn@P and bare Zn. Notably, the nucleation overpotential difference between 3D Zn@P and Zn@P demonstrates that its 3D porous structure benefits the current/electron transport kinetics, which reduces the local circuits and induces Zn^2+^ to undergo uniform deposition. As a result, the average CE value of the 3D Zn@P//Cu battery remained as high as 99.87% after 2000 cycles with a nucleation overpotential as low as 19 mV. It maintained 97.30% capacity after 5000 cycles in a full battery with NH_4_VO_3_ as the cathode, demonstrating excellent stability and long‐cycle performance.

**Figure 7:**
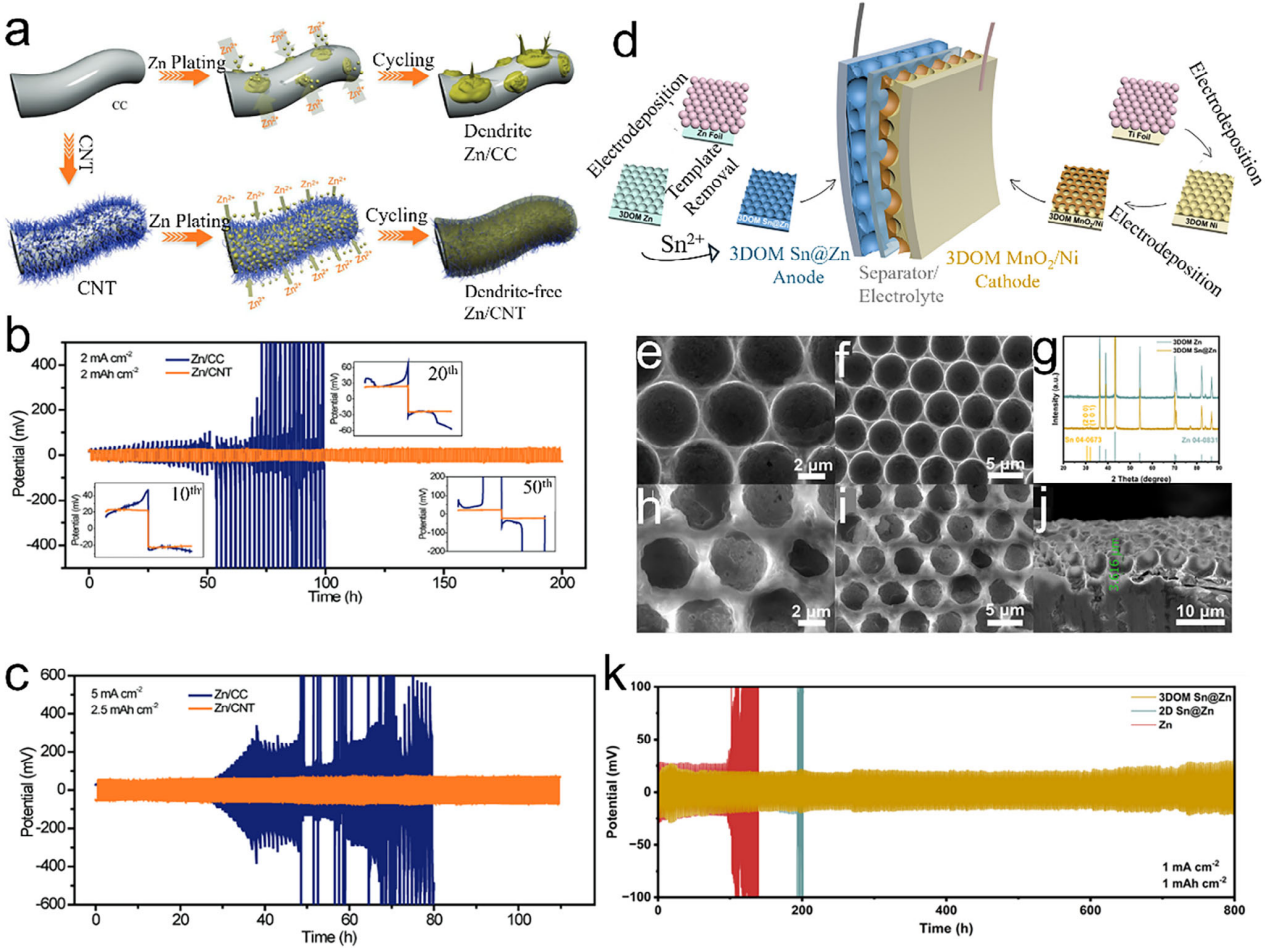
a) Schematic of Zn deposition on CC and CNT. Voltage profile of symmetric cells with Zn/CC and Zn/CNT b) at 2 mA cm^−2^ c) 5 mA cm^−2^. Reproduced with permission.^[^ ^65^ ^]^ Copyright 2019, Wile‐VCH. d) Schematical diagram of the preparation of a 3DOM electrode‐based flexible ZIB. e,f) SEM images of 3DOM Zn. g) XRD of 3DOM Zn and 3DOM Sn@Zn. h–j) SEM images of 3DOM Sn@Zn k) cyclic performance of Zn symmetric cell at 1 mA cm^−2^ and 1 mAh cm^−2^. Reproduced with permission.^[^ ^66^ ^]^ Copyright 2024, American Chemical Society.

Foam Zn materials are also considered a candidate for ZIB anode materials due to their porous structure. For example, Chen et al., plated a copper layer (FZn@Cu) on the surface of foam Zn as an anode for ZIBs by the substitution‐melt‐casting two‐step method.^[^ ^64^ ^]^ The porous sample of FZn@Cu can be seen from the SEM image (Figure 6d), and such a structure can reduce the local current density, which is favorable for the deposition of Zn^2+^ and inhibits the growth of dendrites. Furthermore, the loose structure can also provide a buffer for the volume expansion of the material, which is beneficial to the stability of the anode. In the CV test of the full battery with MnO_2_ as the cathode, the battery with FZn@Cu as the anode has better reaction kinetics and electrochemical performance due to its high current density and smaller voltage polarization (Figure 6e). The project simulated the electric field distribution near the electrode to investigate the role of Cu coating in the FZn@Cu electrode. A surface with Cu nanorod coating (Figure 6f) has a more uniform electric field distribution than bare Zn with tip effect (Figure 6g). The uneven electric field distribution is prevented from causing the gradual local accumulation of Zn^2+^, leading to the formation of dendrites and reducing the anode's cycling stability. Finally, the full battery with FZn@Cu as the anode and MnO_2_ as the cathode can maintain a capacity of 133.90 mAh g^−1^ after 1000 cycles at 1 A g^−1^, showing excellent multiplicative performance and stability. Zeng et al. suggested highly conductive carbon nanotube (CNT) materials to create a 3D framework to attain clean Zn/CNT anodes with electrodeposition.^[^ ^65^ ^]^ The CNT arrays were prepared on flexible carbon cloth (CC) via chemical vapor deposition. 3D CNT frameworks could increase the specific area and decrease the nucleation overpotential. The even distribution of the electric field confirms that Zn^2+^ could be deposited more homogeneously on the complete electrode surface, thus successfully removing zinc dendrites or detrimental by‐products during charging/discharging processes (**Figure** **7**a). The electrochemical performance of the Zn‐CC and Zn‐CNT anodes was examined in a symmetric coin cell utilizing 2 M ZnSO_4_ as an electrolyte. The Zn‐CC//Zn‐CC and Zn‐CNT//Zn‐CNT symmetric cells cycled at various current densities to assess their long‐term cycling stability. The Zn‐CNT//Zn‐CNT symmetric cell indicates a stable voltage profile with a low voltage hysteresis of ≈27 mV at a current density of 2 mA cm^−2^ and a limited capacity of 2 mAh cm^−2^ for 200 h. In comparison, the voltage profile of the Zn‐CC//Zn‐CC displays substantial voltage fluctuation after 50 h and eventually results in cell failure due to dendrite‐induced soft short circuits (Figure 7b). On the contrary, Zn/CNT indicates a remarkable control of the dendrite growth of Zn, which grants Zn/CNT a long cycle life. The significant durability of the Zn/CNT anode compared to the Zn/CC anode can be further demonstrated by cycling at a high current density of 5 mA cm^−2^ (≈35% DOD) (Figure 7c).

Wang et al. fabricated a 60 µm thick 3D ordered macroporous (3DOM) Sn@Zn anode and a stable (3DOM) MnO_2_/Ni cathode, equally having high mechanical flexibility, constructed on a colloidal template method via electrodeposition process (Figure 7d).^[^ ^66^ ^]^ The structured 3D pattern proposed an expanded electrochemically active surface area, aiding an even distribution of local current densities and specific control of Zn deposition and dendrite obstruction. The uneven deposition is constrained by the 3DOM structure at the anode side. The morphology of the electrodeposited 3DOM Zn, in which a structured pattern with a diameter of 4.60 µm is homogeneously arranged on the Zn foil (Figure 7e,f). After the 3DOM Zn is dipped into a SnCl_4_ aqueous solution for 5 min, Sn is shifted onto the sample's surface, as shown by the X‐ray diffraction (XRD) spectra (Figure 7g). Diffraction peaks belonging to (101) and (200) planes of Sn appeared after the displacement reaction to synthesize 3DOM Sn@Zn. After introducing Sn to the Zn surface, the 3DOM structure of the electrode reveals deformation, and the pore size contracts to ≈4.40 µm in diameter (Figure 7h,i). According to the cross‐sectional scanning electron microscopy (SEM) image (Figure 7j), the Sn@Zn hemispherical depth is 3.6 µm. For electrochemical stability, 2D Sn@Zn and Zn morphology after cycling show rigorous deformation at the electrode surface, with large protrusions and irregular accumulation of Zn growth. Comparable results can be noted at 1 mA cm^−2^ and 1 mAh cm^−2^ (Figure 7k). 3DOM Sn@Zn attains regular cycling for 800 h, while 2D Sn@Zn and Zn‐based symmetric cells fail after 200 h, implying that 3DOM structures provide the Zn anode with an extended lifespan.

#### 3.1.2 Nanostructuring of Alloy or Composite Material‐Based Anodes

**Figure 8:**
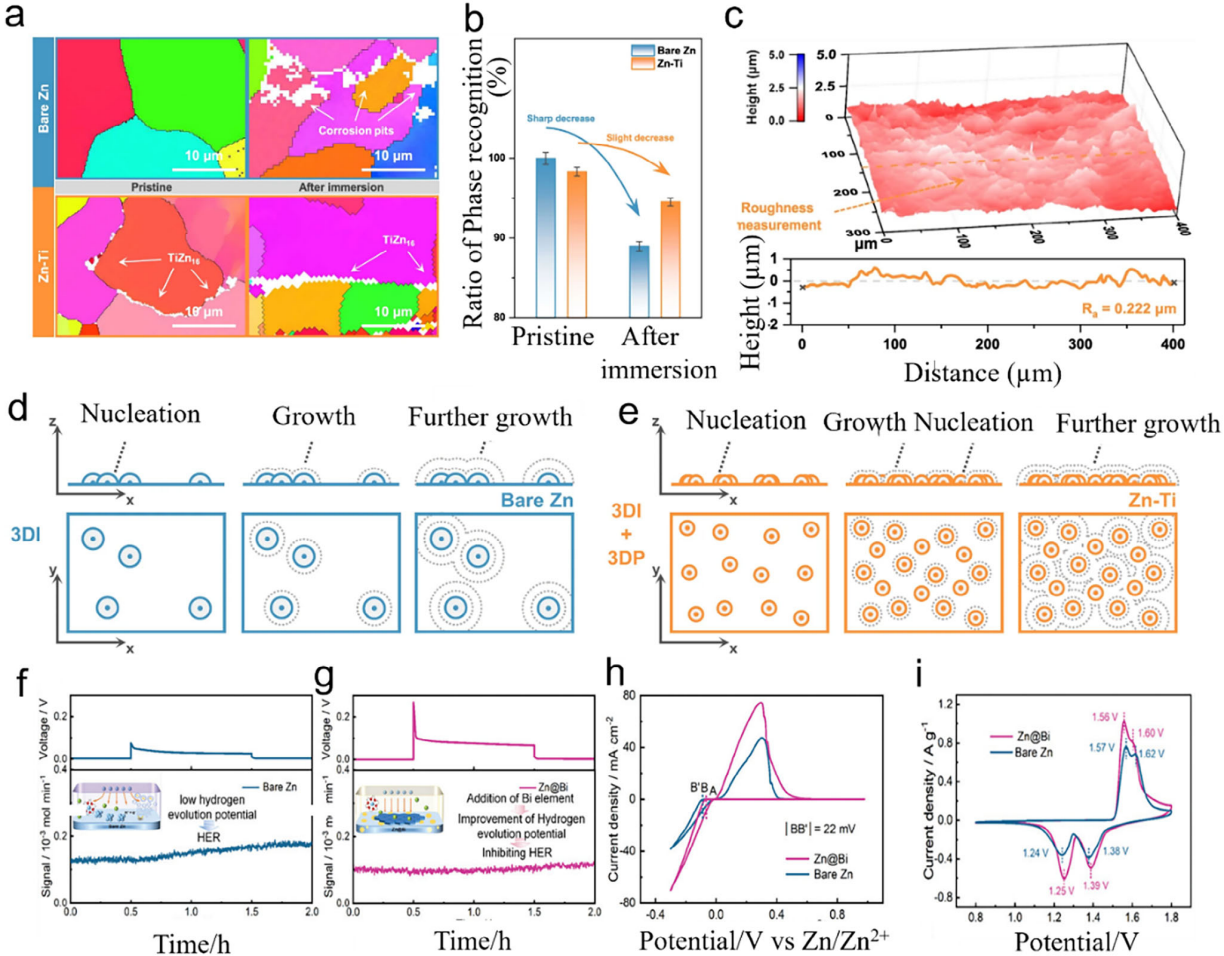
a) EBSD IPF spectra of bare Zn anodes and Zn‐Ti anodes in their original state and after 24 h immersion in ZnSO_4_ electrolyte. b) The corresponding ratio of phase recognition is based on image recognition. c) CLSM imaging of Zn‐Ti alloy corrosion patterns and corresponding surface profiles. Schematic of Zn nucleation growth in d) bare Zn and e) Zn‐Ti alloys. Reproduced with permission.^[^ ^74^ ^]^ Copyright 2023, Springer Nature. In situ DEMS signals and corresponding electrostatic time profiles for f) bare Zn and g) Zn@Bi in symmetric batteries. h) CV curves of bare Zn//Ti and Zn@Bi//Ti batteries. i) CV curves of full batteries with MnO_2_ as the cathode and bare Zn and Zn@Bi as anode. Reproduced with permission.^[^ ^75^ ^]^ Copyright 2024, Wiley‐VCH.

Since applications of ZIBs anodes are plagued by corrosion, HER, and dendrites, researchers have found Zn alloys, including binary (Zn‐Al, Cu‐Zn, and Zn‐Sn, etc.) and ternary Zn alloys (Zn‐Sn‐Pb, Ga‐In‐Zn, Zn‐Li‐Mn, etc) show promise.^[^ ^67^, ^68^, ^69^, ^70^, ^71^, ^72^ ^]^ Moreover, the properties of Zn alloy anodes can be changed through strategies such as modulating the phase components and phase distribution in the alloys, which can effectively improve the anode's ability to circulate and store charge in ZIBs. The reported Zn alloys can be classified into bulk‐phase Zn alloy and surface Zn alloys (i.e., alloy films prepared on the surface of Zn anodes).^[^ ^73^ ^]^ Coronation of metals starts at grain boundaries (GB), and modification of GB is significant, especially in grain boundary engineering (GBE). For example, Zhang et al., modulated the GB of a Zn metal base by constructing a Zn‐Ti alloy body and made it act as an anode for ZIBs to inhibit corrosion and enhance cyclability.^[^ ^74^ ^]^ Electron Backscattered Diffraction (EBSD) analysis was used to corroborate the important role of GBE in suppressing streamlined corrosion (**Figure** **8**a). The EBSD inverse pole figure (IPF) plots of bare Zn and Zn‐Ti alloys are clear in the pristine state, with the unidentifiable phase in the Zn‐Ti alloy being TiZn_16_ (solid solutions and intermetallic compounds) IMC. After immersing in the ZnSO_4_ electrolyte, the bare Zn appears to corrode visibly along the GBs and spreads to the interior of the grains. The phase identification rate decreased from 100% to 89%, while the Zn‐Ti alloy only decreased from 98% to 95% (Figure 8b). In addition, confocal laser scanning microscopy (CLSM) imaging (Figure 8c) shows that the immersed Zn‐Ti alloy maintains a relatively flat appearance, demonstrating its excellent corrosion resistance. After the Zn nuclei appeared on the nucleation sites of bare Zn, the Zn nuclei would not continue to grow on the new nucleation sites due to an elevation in the nucleation barrier but gradually accumulated on the original Zn nuclei, leading to the growth of dendrites (Figure 8d). However, due to the Zincophilic nature of TiZn_16_ IMC, it can provide more Zn nucleation sites, induce the uniform deposition of Zn^2+^, avoid the growth of dendrites (Figure 8e), and enhance the stability of ZIBs with Zn‐Ti as an anode. Finally, in electrochemical tests, the Zn reversibility of the Zn‐Ti alloy anode was as high as 99.85% in 4000 cycles, and the number of cycles of the full battery with NH_4_V_4_O_10_ as the cathode was ≈3500, which demonstrated the full potential for application. Yang et al., synthesized Zn@Bi anodes using bismuth, a homolog of phosphorus, by the melting method to enhance the recyclability of Zn anodes.^[^ ^75^ ^]^ From the in situ differential electrochemical mass spectrometry (DEMS) tests of bare Zn and Zn@Bi in Na_2_SO_4_ solution indicated that bare Zn spontaneously produces H_2_ gas and undergoes HER (Figure 8f), but no H_2_ signals could be detected on Zn@Bi (Figure 8g), which proves the ability of Zn@Bi to inhibit HER.

**Figure 9:**
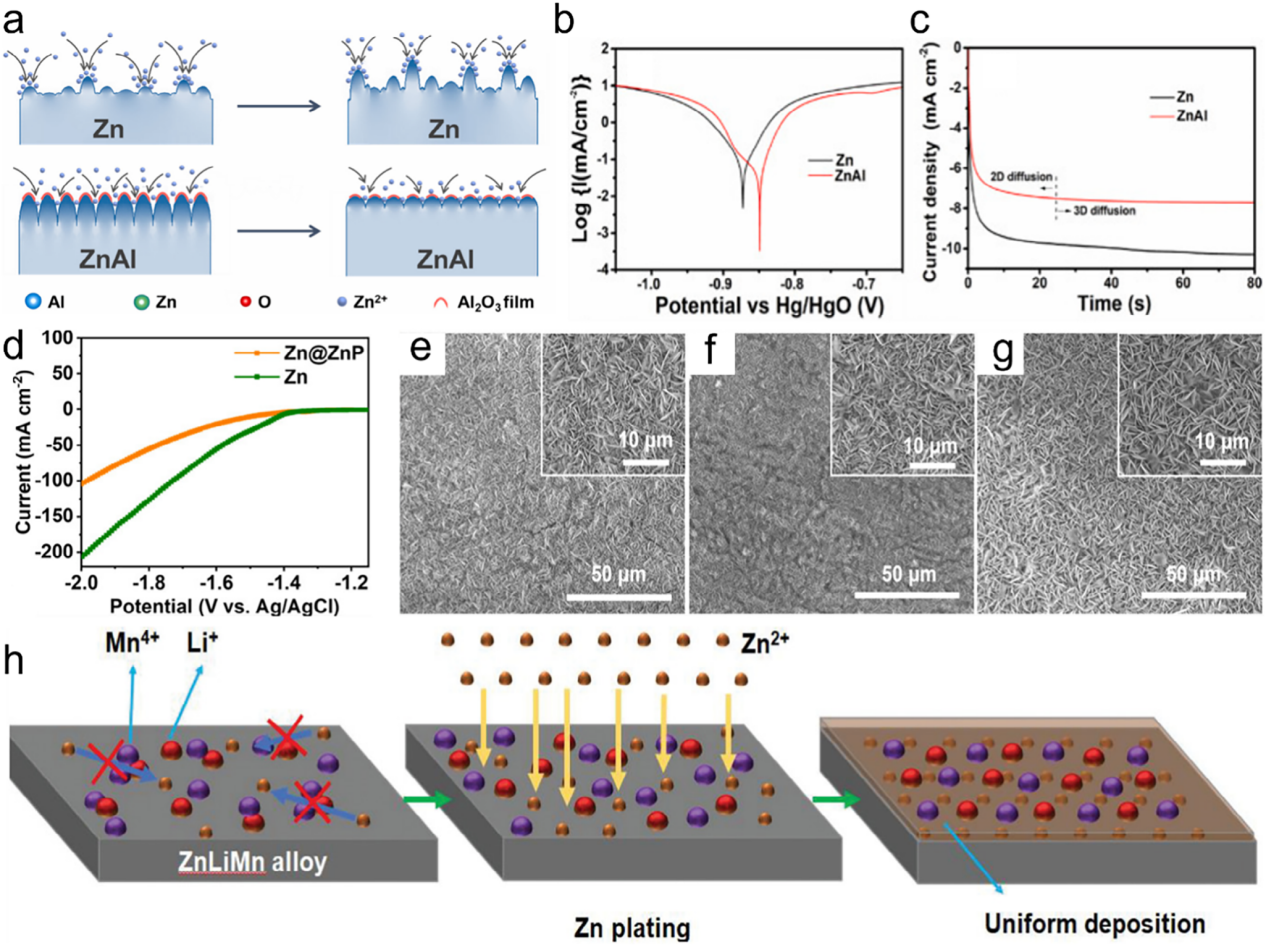
a) Schematic representation of Zn^2+^ deposition on bare Zn and Zn‐Al alloy anodes. b) Linear polarization curves of bare Zn and Zn‐Al alloy in 1 M Zn (CF_3_SO_3_)_2_. c) Current‐time curves of bare Zn and Zn‐Al alloys at −150 mV overpotential. Reproduced with permission.^[^ ^67^ ^]^ Copyright 2023, Elsevier. d) LSV curves of HER test for Zn and Zn@ZnP in 1 M Na_2_SO_4_ solution. e–g) SEM images of Zn@ZnP anode with different capacity limits at a current density of 1 mA cm^−2^. Reproduced with permission.^[^ ^76^ ^]^ Copyright 2023, Elsevier‐VCH. h) Schematic flow diagram of Zn^2+^ deposition on ZnLiMn alloy anode. Reproduced with permission.^[^ ^72^ ^]^ Copyright 2022, Elsevier‐VCH.

The nucleation overpotential of Zn@Bi/Ti is 22 mv lower than that of the Zn/Ti battery (Figure 8h), indicating that Zn@Bi is less likely to make dendrite growth compared to the bare Zn anode. In addition, according to the CV curve of the full cell with MnO_2_ as the cathode and bare Zn and Zn@Bi as the anode (Figure 8i), the Zn@Bi anode has a higher peak current and narrower voltage difference, which indicates higher electrochemical activity and lower polarization. Finally, in the cyclability test, the Zn@Bi//MnO_2_ battery could be cycled 1700 times at 1.20 A g^−1^ and maintained a specific capacity of 119.30 mAh g^−1^. Xiao et al. reported a Zn‐Al alloy that improves the cyclability of ZIBs by forming Al_2_O_3_.^[^ ^67^ ^]^ During the cycling process, Al in the anode of the Zn‐Al alloy reacts with O_2_ in the electrolyte to form Al_2_O_3_, which prevents Zn from reacting with O_2,_ leading to passivation and preventing the continued dissolution of Al. The formation of Al_2_O_3_ contributes to the formation of Zn “grooves”, inducing uniform deposition of Zn^2+^ on these grooves (**Figure** **9**a), effectively inhibiting uncontrolled 2D diffusion of Zn^2+^ and hindering the formation of dendrites. (Figure 9b) shows the linear polarization curves of bare Zn and Zn‐Al alloy in 1 M Zn(CF_3_SO_3_)_2_, where the corrosion potential of the Zn‐Al alloy rises from −0.87 to −0.84 V and the corrosion current decreases, which illustrates that it has a smaller corrosion current and has a better corrosion resistance than bare Zn. The shorter 2D diffusion of Zn^2+^ on the electrode surface of Zn‐Al alloy than bare Zn (Figure 9c), can effectively inhibit the growth of dendrites. Finally, the Zn‐Al//V_6_O_13_ full battery was cycled 1000 times at 3 A g^−1^ and maintain 95% capacity (specific capacity of 280 mAh g^−1^). Ma et al. Also achieved high cycling stability of Zn anodes by growing a 3D structure Zn‐P alloy protective layer in situ on the surface of Zn anodes by etching.^[^ ^76^ ^]^ In the LSV test of the Zn@ZnP anode and Zn anode (Figure 9d), the HER current density of the Zn@ZnP anode is much lower than that of the Zn anode in a relatively wide voltage range, which proves the better inhibition of hydrogen precipitation behavior. In situ SEM showed no obvious dendrite growth on the surface of the Zn@ZnP anode under different capacity limitations, maintaining the uniform deposition of Zn^2+^ (Figures 9e–g). Exhibiting such a stable behavior can be attributed to the fact that the 3D structure of the Zn@ZnP anode has a large specific surface area, which provides more Zn^2+^ deposition sites, a uniform electric field, and a smooth surface, which can also inhibit the growth of dendrites. The final symmetric battery with Zn@ZnP electrodes exhibits a long‐term cycle life exceeding 1260 h at a current density of 10 mA cm^−2^. The full battery, comprising a Zn@ZnP anode and a MnO_2_‐based cathode, demonstrates a high discharge capacity of 145 mAh g^−1^ after 500 cycles at a current density of 1000 mA g^−1^.

Ternary alloy Zn anodes have also been reported to adjust the movement of Zn^2+^ to inhibit dendrite formation. For example, Xie et al., prepared a ZnLiMn ternary alloy as a competitor for the anode in ZIBs.^[^ ^72^ ^]^ Since Zn^2+^ undergoes 2D diffusion on the surface of the bare Zn anode during plating/stripping until it encounters an existing nucleation site, this uncontrolled diffusion leads to the growth of dendrites. The formation of ZnO by‐products with O_2_ will passivate the deposited Zn. On the contrary, since the standard equilibrium potentials of Li^+^/Li and Mn^2+^/Mn are lower than that of Zn^2+^/Zn. So, Li and Mn in the ZnLiMn ternary alloy anode preferentially react with O_2_ in the system to form cations, such as Li^+^ and Mn^4+^, which prevents the passivation of Zn, and the electrostatic shielding generated by these cations prevents Zn^2+^ from diffusing into the existing nucleation sites. The 2D diffusion of the nucleation sites induces the uniform deposition of Zn^2+^. It avoids the growth of dendrites (Figure 9h), which enhances the cyclability of the battery with ZnLiMn ternary alloy as the anode. Moreover, the final ZnLiMn ternary alloy anode has excellent mechanical properties, and the assembled ZnLiMn/MnO_2_ full battery can maintain 96% capacity after 400 cycles at 1C. Overall, working on structures of the Zn anode enhances the efficiency and stability of the ZIBs by reducing Zn dendrites, mitigating HER, and lowering corrosion. However, the formation of the protective layer through interface modification is also an important and innovative strategy. Here, **Table** **1** summarizes the role of the composite material‐based anode in ZIBs.

**Table 1:** Function of the composite material‐based anode.

Anode	Anode function	Electrolyte	Current density [mA cm^−2^]	Capacity [mAh g^−1^]	Cathode	Cycle number	Refs.
3DP‐NC@Zn	Facilitates ion diffusion and provides ion replenishment.	1 M ZnSO_4_	5	199.9	VO_2_	NA	[82]
3DP‐PC/SiOC@Zn	Improved electrical conductivity and active sites to guide uniform nucleation of Zn2+.	3 M ZnSO4	0.5	67	V_2_O_5_/C	2435	[83]
3DP‐BU@Zn	A dual gradient electron/ion flux was established with simultaneous, enhanced electron transfer and ion diffusion.	1 M Zn(CF_3_SO_3_)_2_	1	252.8	VO_2_	Over 500	[84]
Sn@3D‐Zn	Providing more zinc nucleation sites with lower deposition energy barriers encourages lateral growth of zinc electrodeposits along Zn(002).	2 M ZnSO_4_	1	300.8	PANI‐V_2_O_5_	Over 2000	[85]
Zn@CC‐CNF	Achieves uniform zinc deposition with a low nucleation barrier and mitigates side reactions.	2 M ZnSO_4_	1	200	MnO_2_	Over 300	[86]
3DP‐UiON‐PHC@Zn	Improved ion channels for efficient Zn2+ ion transport.	3 M ZnSO_4_	0.4	81.2	3DP‐VOC@Al	260	[87]
3D‐VG	The VS4 phase of the anode promotes rapid diffusion control dominating the electrochemical kinetics.	1 M Zn(CF_3_SO_3_)_2_	1	468	V_6_O_13_	Over 2000	[88]
ZnOHF NWs@Zn	Provide zincpphilic properties and homogenize the electric field	2 M ZnSO_4_ with 0.1 M MnSO_4_	5	72.8	MnO_2_@CC	Over 600	[89]
ss‐ZnP	Enhancing charge transfer, mitigating bulk effects, and homogenizing interfacial electric fields	2 M ZnSO_4_ + 0.1 M MnSO_4_	1	300	NH_4_V_4_O_10_	500	[90]
Zn@N‐VG@CC	The Zn nucleation overpotential is reduced and the 3D structure results in a uniform electrical distribution.	2 M ZnSO_4_ and 0.4 M ZnSO_4_	2	252	MnO_2_@N‐VG@CC	Over 300	[91]
ZnAl@Cu‐mesh	Restraining the lateral diffusion of zinc ions	3 M Zn(CF_3_SO_3_)_2_	2	1	V_2_O_5_	Over 2000	[92]
ZnBi	Bi inhibits HER	2 M ZnSO_4_ and 0.1 M MnSO_4_	5	186	V_2_O_5_	Over 1000	[93]
F‐CRZF@Zn	Promotes ionic migration while significantly lowering the nucleation potential of Zn; enhances the HER overpotential on Zn and supports homogeneous Zn deposition	2 M ZnSO_4_	10	548	VS_2_/MXene	Over 5000	[94]
In@Zn@In	Rapid penetration of electrolytes, ample electron/ion transport channels, and stable structure.	2 M ZnSO_4_·7H_2_O and 0.2 M MnSO_4_·H_2_O	1.8	123	Mn_2_O_3_	Over 2000	[95]
Zn88Al12	Promoting zinc stripping from precursor alloys to generate core/shell aluminum/semi‐alumina interlayer nanopatterns in situ to guide zinc growth	2 M ZnSO_4_ and 0.2 M MnSO_4_	0.5	300	K_0.12_MnO_2_	Over 5000	[96]

### 3.2 Interface Engineering

Interface engineering is key for regulating the deposition behavior of Zn ions and inhibiting the formation of Zn dendrites and side reactions in ZIBs.^[^ ^77^ ^]^ A stable electrode/electrolyte interface ensures homogenous charge density and higher charge transfer, which prevents electrode dissolution, etc. The direct contact of the electrode with electrolyte generates a surface interphase layer commonly known as solid electrolyte interphases (SEI), which is formed by electrolyte reduction on the electrode surface and is critical in determining the stability of the interface. The ideal SEI should have high Zn ionic conductivity, electronically insulating ability, high chemical and thermal stability, and high mechanical strength.^[^ ^78^, ^79^, ^80^ ^]^ The interface chemistry is typically regulated by the composition of the electrolyte, which can be explained by Goodenough's electron‐energy model.^[^ ^81^ ^]^

**Figure 10:**
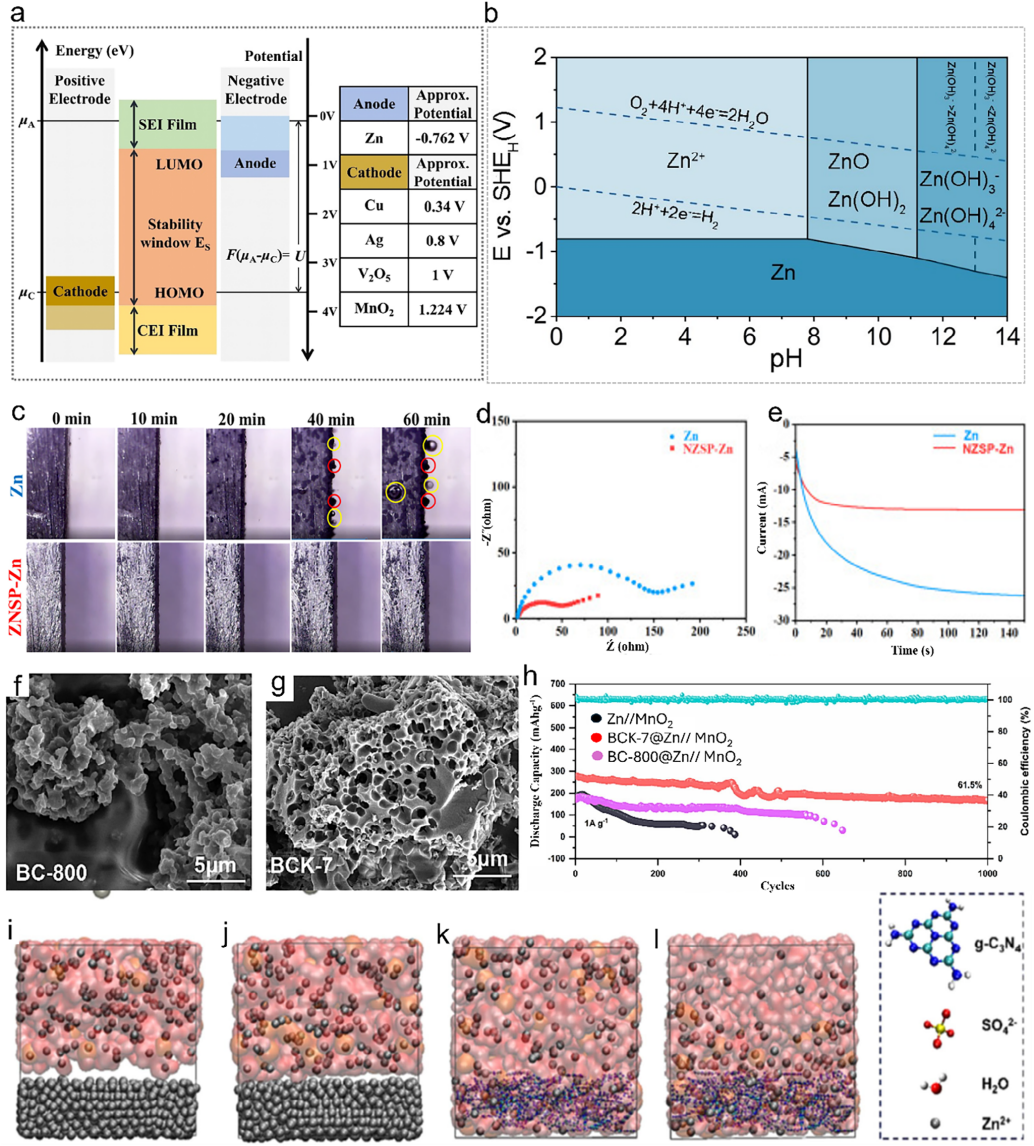
a) Illustration of band energy electrolytes. Reproduced with permission.^[^ ^81^ ^]^ Copyright 2022, Springer Science. b) Pourbaix diagram of Zn in aqueous solution. Reproduced with permission.^[^ ^105^ ^]^ Copyright 2023, The Royal Society of Chemistry. c) In situ optical micrograph images show cross‐sections of Zn and NZSP‐Zn with a current density of 10 mA cm^−2^ and different deposition times. Red circles indicate Zn protrusions, and yellow circles indicate hydrogen bubbles. d) EIS test of Zn and NZSP‐Zn anodes in symmetric batteries before cycling. e) Zn deposition behavior of Zn and NZSP‐Zn anodes in symmetric batteries. Reproduced with permission.^[^ ^100^ ^]^ Copyright 2023, Elsevier. Scanning electron microscopy (SEM) image of f) BC‐800 and g) BCK‐7. h) Cycling performance of Zn, BC‐800@Zn, and BCK‐7@Zn in MnO_2_ cathode full battery.^[^ ^102^ ^]^ MD simulations of i) Zn without electric field, j) and under 0.50 V nm^−1^ electric field strength. MD simulations of k) g‐C_3_N_4_ without electric field strength l) and under 0.50 V nm^−1^ electric field. Reproduced with permission.^[^ ^103^ ^]^ Copyright 2023, Elsevier.

To obtain a thermodynamically stable interphase, the electrolyte's lowest unoccupied molecular orbital (LUMO) must be higher than the Fermi level of the anode (µ_A_). In contrast, the highest occupied molecular orbital (HOMO) of the electrolyte must be lesser than the Fermi level of the cathode (µ_C_). However, the potential of the electrode is usually more than the electrochemical window of electrolytes until interphases (SEI or CEI) cease electron shifting among LUMO and HOMO of the electrode and electrolyte (**Figure** **10**a).^[^ ^97^ ^]^ When the electrode potential is in between LUMO and HOMO, there is no sudden reaction at the interface, so a durable electrolyte‐electrode interface (EEI) can be attained, outside these limits, the electrolyte oxidizes at the electrodes. Whereas the electrochemical stability windows (ESW) of aqueous electrolytes are narrow due to oxygen evolution reactions (OER) and HER. The increase of the pH value in aqueous solutions, Zn can be oxidized to dissolved forms of Zn^2+^, HZnO^2−^, Zn (OH)_2_, and ZnO_2_^2−^ (Figure 10b).^[^ ^49^ ^]^ When the pH is <4, Zn has a high solubility and can be simply dissolved as Zn^2+^. At 5 < pH < 8, compared with an effective acidic solution, the dissolution of Zn becomes reasonably slow due to the high overpotential and low corrosion activity. Under slightly alkaline solutions at 8 < pH < 10, irreversible byproducts are formed during the charge/discharge process on the surface of Zn anodes (e.g., Zn (OH)_2_, ZnO). At pH > 11, Zn solubility reduces again, and zincate ions (e.g., Zn (OH)_4_^2−^) start to originate.

The electrode‐electrolyte interface (SEI layer) can be designed to protect the electrode material from direct contact with the electrolyte, reduce side reactions, and increase the stability of the battery. Several techniques have been investigated in this regard, including inner Helmholtz layer modulation, polymer or organic coating, Zn^2+^ cation solvation structure modification, and ceramic or inorganic coatings. The utilization of artificial SEI, where the electrodes are coated with a thin layer before integration in the battery configuration, as well as the tuning of electrolyte chemistry to stabilize the electrode/electrolyte interface, has been widely reported. In this section, we will discuss the artificial SEI and in situ SEI (electrolyte additive), explaining the different strategies to overcome the challenges mentioned above and increase the performance of Zn anode in ZIBs.

#### 3.2.1 Artificial Solid Electrolyte Interphase (ASEI)

The growth of dendrites can be prevented by constructing a physical protective layer between the Zn metal anode and the electrolyte, which avoids direct contact with the electrode to control ion migration, restrain side reactions, and limit HER.^[^ ^98^ ^]^ The protective ASEI layer can greatly improve the interfacial stability and cycling durability of the Zn electrode by homogenizing the electric field and facilitating the desolvation of the metal ions at the electrode/electrolyte interface.^[^ ^99^ ^]^ Several materials have been proposed as ASEI, and the choice of ideal coating material depends on the ability to stabilize the interface between electrodes and electrolytes. For ASEI, inorganic compounds, organic and polymer‐based materials, carbon‐based materials, ionic liquids, gel electrolytes, and hybrid (organic‐inorganic) materials have been widely utilized.

Among the above materials, certain metals possess excellent electrical conductivity and zincophilic properties, making them effective materials for anodic face engineering. For example, Lu et al. fabricated a Na_3_Zr_2_Si_2_PO_12_ (NZSP) protective layer on the Zn anode by the drop injection method to inhibit the growth of dendrites and mitigate the side reactions and hydrogen precipitation phenomena, which enhances the cyclability of the Zn anode.^[^ ^100^ ^]^ Figure 10c shows the Zn deposition behavior and gas evolution on Zn and NZSP‐Zn anode at 10 mA cm^−2^. After 40 min of cycling, dendrite growth (red circles) and hydrogen precipitation (yellow circles) were observed on the Zn anode surface. In contrast, no significant dendrite growth and hydrogen precipitation were observed on NZSP‐Zn. Moreover, in the electrochemical test of the symmetric battery, the charge transfer resistance of the NZSP‐Zn anode was lower than that of the Zn anode both before and after cycling, which proves the better transfer kinetics of the NZSP‐Zn anode (Figure 10d). In addition, the current on the Zn anode continues to increase after 150 s, suggesting a slower 2D transfer rate, leading to rough deposition and dendritic growth (Figure 10e). Whereas the NZSP‐Zn anode promoted a faster 2D transfer rate, followed by stable 3D diffusion within 20 s, indicating that the localized reduction of Zn^2+^ at the interface prevented the accumulation of dendritic growth. Consequently, the symmetrical battery based on NZSP‐coated Zn demonstrated stable cycling for 1360 h at a current density of 0.50 mA cm^−2^ and a cycling capacity of 0.50 mAh cm^−2^. It also maintains stability for 1000 h at higher current densities of 5 mA cm^−2^ and a 2 mAh cm^−2^ cycling capacity.

Carbon‐based materials have been widely utilized in the protective layer engineering of Zn anodes. These materials offer inherent advantages, including high electrical conductivity, wide availability, low cost, environmental friendliness, and high stability. In addition, carbon materials provide a higher number of nucleation sites, which can be used as an effective protective layer for Zn anodes.^[^ ^101^ ^]^ Therefore, efforts have been made to modify Zn anodes using carbon‐based materials to improve the electrochemical performance of ZIBs. For instance, Zhu et al. developed porous biomass carbon (BCK) as a protective layer for Zn anodes.^[^ ^102^ ^]^ The obtained BCK was first calcined in a tube furnace at 800 °C, labeled as BC‐800 (Figure 10f), and then activated to biomass char BCK‐7 (Figure 10g) by hydrothermal reaction with 7% KHCO_3_ solution. From the SEM images, BCK‐7, after KHCO_3_ activation, has BCK with more pores than BCK‐800 and, therefore, has a larger surface area. Due to the abundance of oxygen‐containing functional groups and the larger specific surface area of BCK‐7, it can provide sufficient nucleation sites for Zn^2+^, which enhances the transport kinetics of Zn^2+^ and homogenizes the surface electric field of the Zn anode. Therefore, even after 1000 cycles at the current density of 1 A g^−1^, the capacity of the full‐cell battery can still reach 166.50 mAh g^−1^, with a capacity retention rate of 61.50%, which is significantly higher than both bare Zn and BC‐800‐based full cell battery (Figure 10h).

Polymers also play an important role in Zn anode surface engineering to improve ZIBs performance. For instance, Pan et al. constructed a protective layer of g‐C_3_N_4_ (graphitic carbon nitride) on Zn anode surfaces by simply scraping and utilizing its Zincophilicity to inhibit side reactions, HER, and dendritic phenomena.^[^ ^103^ ^]^ The authors further performed molecular dynamics (MD) simulations (Figure 10i–l) to investigate the interactions between Zn^2+^ and metal Zn and g‐C_3_N_4_ in the ZnSO_4_ electrolyte. The free state Zn^2+^ encapsulated by water molecules in (Figure 10i) proves that the bare Zn surface is not zincophilic. On the contrary, to keep the system energy low, most of the Zn^2+^ in the electrolyte formed a complex state with g‐C_3_N_4_ molecules on the surface of the g‐C_3_N_4_ layer, suggesting that g‐C_3_N_4_ has zincophilic properties (Figure 10j). When an electric field strength of 0.50 V nm^−1^ was applied, the free Zn^2+^ in Zn_2_SO_4_ clustered with the g‐C_3_N_4_ molecules (Figure 10k), then there was a significant reduction of free Zn^2+^ in the electrolyte, unlike the movement of free Zn^2+^ toward bare Zn alone, which was not significantly reduced in the electrolyte (Figure 10l). Thus, g‐C_3_N_4_ can reduce the nucleation energy of Zn^2+^ and induce deposition, thus inhibiting dendrite growth. In the final symmetric battery test, the g‐C_3_N_4_@Zn anode achieved a longer cycling time of 2900 h and a higher stability of 1000 cycles in the g‐C_3_N_4_@Zn//V_3_O_7_ H_2_O full battery.

Similarly, using the Langmuir‐Blodgett method, Liu et al. designed a uniform nitrogen‐rich and highly conductive carbon protective layer on the Zn anode. This protective layer is corrosion‐resistant in aqueous electrolytes and provides abundant nucleation sites for Zn^2+^, which facilitates a conducive, uniform deposition of Zn^2+^. As a result, the CN‐Zn||CN‐Zn symmetric battery has a high capacity (5 mAh cm^−2^) with a reversible plating/stripping life of more than 1300 h at an ultra‐low overpotential (24 mV) and a cycling capacity of 1 mAh cm^−2^. In addition, the CN‐Zn ||MnO_2_ symmetric battery achieves excellent electrochemical performance with a discharge capacity of 116 mAh g^−1^ at 5 mA and outstanding stability.^[^ ^104^ ^]^

**Figure 11:**
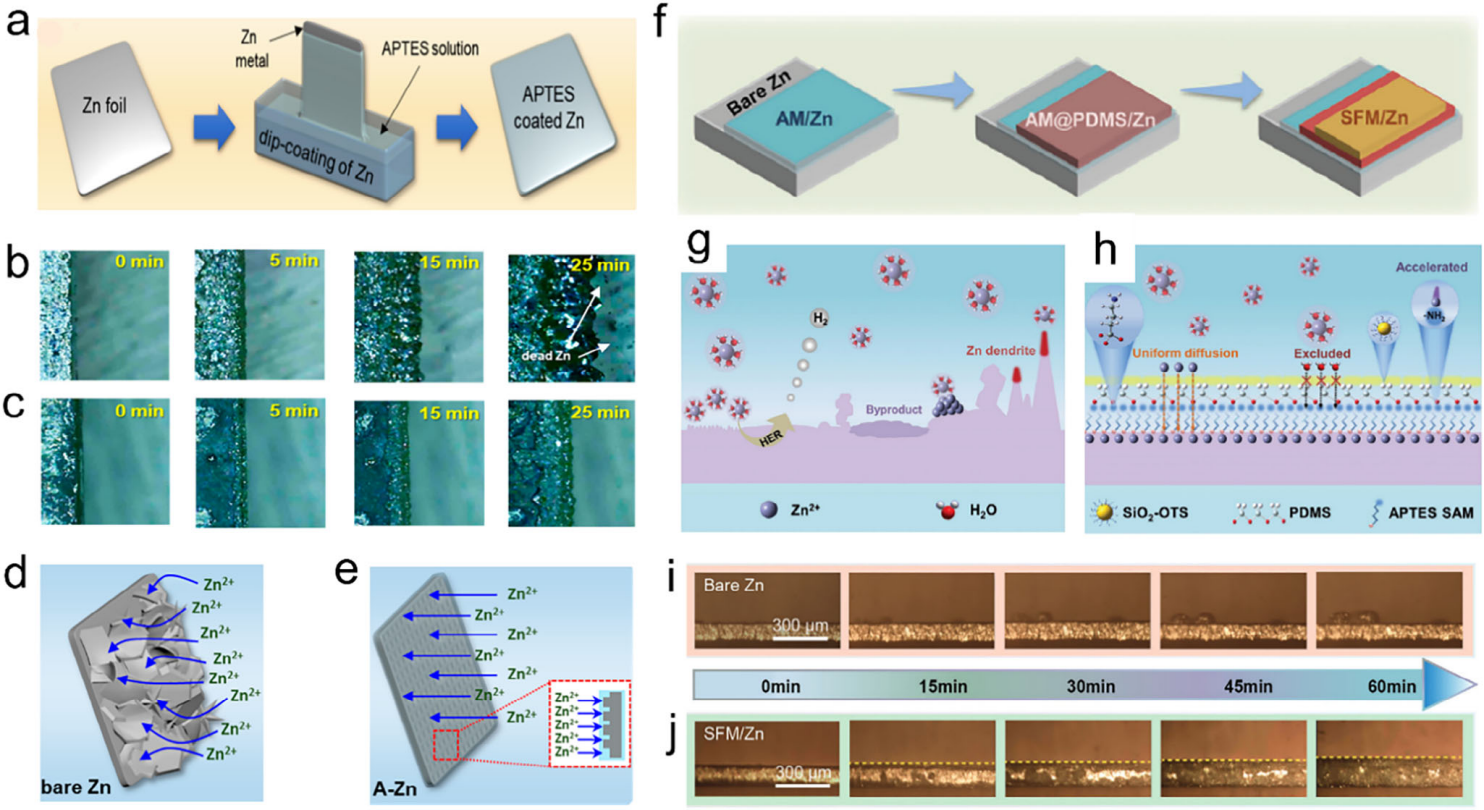
a) Schematics of ASEI coating on Zn anode and b) In situ optical microscopy without ASEI c) In situ optical microscopy representing artificial coating. Schematics of Zn surfaces illustrating Zn deposition performance d) bare Zn, e) APTES coated Zn. Reproduced with permission.^[^ ^106^ ^]^ Copyright 2021, American Chemical Society. f) Sequence of hydrophobic three‐layered SFM/Zn anode. Schematics Zn deposition g) on bare Zn h) on SFM/ZN with 2 M ZnSO_4_. In situ optical microscopy at 3 mA cm^−2^ for 1 h i) on bare Zn j) SFM/ZN anode. Reproduced with permission.^[^ ^107^ ^]^ Copyright 2024, Wiley‐VCH.

As already alluded, protective layers of organic matter for Zn anodes have also been reported. Zhang et al., constructed a Zincophilic homogeneous Zn tartrate layer on a Zn anode to prevent dendrite growth by an ultrasonic coating method that can remove adsorbed H_2_ and weakly adherent layers generated during processing due to its excellent corrosion resistance and uniform deposition of Zn^2+^.^[^ ^31^ ^]^ Consequently, it achieved more than 1300 h lifetime at a high current density of 2 mA cm^−2^ and maintained 82.70% capacity after 500 cycles at 1 A g^−1^ in a full battery with MnO_2_ as the cathode. Understanding the morphology of Zn deposition is significantly involved in the surface hydrophilicity of the Zn anodes. Meanwhile, increased surface hydrophilicity allows smoother Zn^2+^ flux through the surface of Zn, resulting in homogeneous zinc plating and nucleation without dendrite growth. Sung Hyun et al. prepared a silane‐based hybrid material to develop a hydrophilic ASEI for surface protection of Zn‐anode using (3‐aminopropyl)triethoxysilane (APTES) via dip coating method with a thickness of ≈500 nm (**Figure** **11**a).^[^ ^106^ ^]^ Deposition of Zn was observed through in situ optical microscopy at a high current density of 30 mA cm^−2^. After 15 min, inhomogeneous deposition of Zn on the bare electrode was observed with the formation of dendrites and dead Zn after 25 min, which no longer took part in the further cycling process (Figure 11b). Meanwhile, APTES‐coated Zn metal showed remarkable performance with smooth surface morphology, exhibiting even coating after 25 min (Figure 11c).

Initially, the Zn^2+^ gets placed on the Zn metal anode, making nuclei at thermodynamically ideal sites and spreading them into protrusions on the surface of uncoated Zn. These protrusions construct a durable electrical field, where Zn^2+^ chooses to accumulate instead of on the smooth areas. Accordingly, the flaky formation of Zn dendrites is unevenly produced. It covers the surface of uncoated Zn, which can penetrate across the separator and ultimately activate an unpreventable internal short circuit, causing the battery failure (Figure 11d). In contrast, the ASEI layer develops a hydrophilic coat, which assures homogeneous spreading of solvated Zn^2+^ on coated Zn. The ASEI layer limits the movement and transportation of Zn^2+^ owing to polysiloxane and hydrocarbon chains received from APTES, successfully blocking out widespread dendrite formation on the surface of Zn anodes (Figure 11e). Consequently, ASEI enhanced the homogeneous deposition of Zn^2+^, increased electrolyte wetting, minimized HER, surged ions transportation, and enhanced cyclic performance in ZIBs.

Based on the above results, Zn deposition stability for APTES‐coated Zn showed exceptional performance at a higher current density of 20 mA cm^−2^ with 5 mAh cm^−2^ areal capacity for 600 h and 99.31% of Zn utilization after 900 cycles at a current density of 2 mA g^−1^ and 1.60 mAh cm^−2^ areal capacity. Additionally, A‐Zn//MnO_2_ full batteries at a current density of 1000 mA g^−1^ maintained a high capacity of 189 mAh g^−1^ even after 3000 cycles. Moreover, in terms of hydrophobic (ASEI), Zhou et al., worked on polydimethylsiloxane (PDMS) and trimethoxy(octadecyl)silane (OTS) by redesigning particles of nano silicon dioxide, which helps to break off H_2_O‐based side reactions.^[^ ^107^ ^]^ Here, by resembling the hydrophobic nature of the lotus leaf, the surface alteration strategy is to optimize the Zn anode by developing a water‐repellent and flexible ASEI. So, a superhydrophobic and flexible ASEI on the Zn anode is fabricated by a three‐layered arrangement that incorporates the required properties. The first layer is the monomolecular layer made by triethoxy‐3‐aminopropyl silane; the next one is a very flexible layer constructed through polydimethylsiloxane (PDMS), while the last layer is a superhydrophobic layer made of trimethoxy(octadecyl)silane (OTS) modified nano silicon dioxide (SiO_2_) and PDMS (all layers symbolized as SFM) (Figure 11f). The assembly of this ASEI merges the self‐assembly of molecules, the squeegee coat process, and the air spraying procedure. Extremely elastic PDMS inhibits polymer breakdown during the dynamic progression of Zn^2+^ and proceeds as a uniform Zn^2+^ diffusion layer throughout Zn^2+^ plating/stripping.^[^ ^108^ ^]^ The OTS‐altered SiO_2_ particles (symbolized as SiO_2_‐OTS and SiO_2_ particles are differentiated by their hydrophobicity. Through spraying SiO_2_‐OTS particles on the surface of PDMS, a hydrophobic layer with a micro‐nano composite lumpy structure deters free H_2_O molecules in the electrolyte, avoiding corrosion on the Zn‐metal anode. The irregular deposition of Zn^2+^ on the Zn anode generates dendrite growth (Figure 11g). This problem is further intensified due to higher current densities. Water molecules in the electrolyte can experience a reduction reaction, leading to the production of H_2_ gas and OH^−^. Mixing OH^−^ and Zn^2+^ with sulfates in the electrolyte can generate Zn‐sulfate, as shown in Equations (9) and (10).

(9) $$2H_{2}O+2e^{-}\to 20H^{-}+H_{2}\left(\mathrm{gas}\right)$$

(10) $$4Zn^{2+}+6OH^{-}+{\mathrm{SO}}_{4}^{-2}+H_{2}O\to Zn_{4}SO_{4}{\left(\mathrm{OH}\right)}_{6}\cdot H_{2}O$$

The hydrophobic (SFM/Zn) layer with SiO_2_–OTS and PDMS is a protective barrier that stops free water molecules, makes uniform diffusion, and inhibits HER. The ASEI with vastly flexible polymer supports the even deposition of Zn^2+^ and efficiently controls the irregular current density distribution (Figure 11h). In situ optical microscopy revealed that the uncoated Zn anode at 3 mA cm^−2^ suffered dendrites and intense HER after 15 min of the Zn deposition, which is escorted through the production of byproducts that undermine the cycling life of the battery (Figure 11i). The SFM/Zn‐coated anode stayed smooth and homogenous under the same conditions even after 60 min. The formation of ASEI showed a reliable and regular deposition of Zn^2+^ (Figure 11j). Subsequently, the symmetric batteries (SFM/Zn||SFM/Zn) showed lasting and reversible Zn plating/stripping at ultrahigh current density (45 mA cm^−2^) and low current density (0.20 mA cm^−2^). In the case of Zn‐vanadium full‐cell (SFM/Zn||NH_4_V_4_O_10_), the CE is 100%, and cyclic stability is (135.50 mAh g^−1^ after 500 cycles at 5 A g^−1^ and 173.20 mAh g^−1^ after 1000 cycles at 2 A g^−1^).

**Figure 12:**
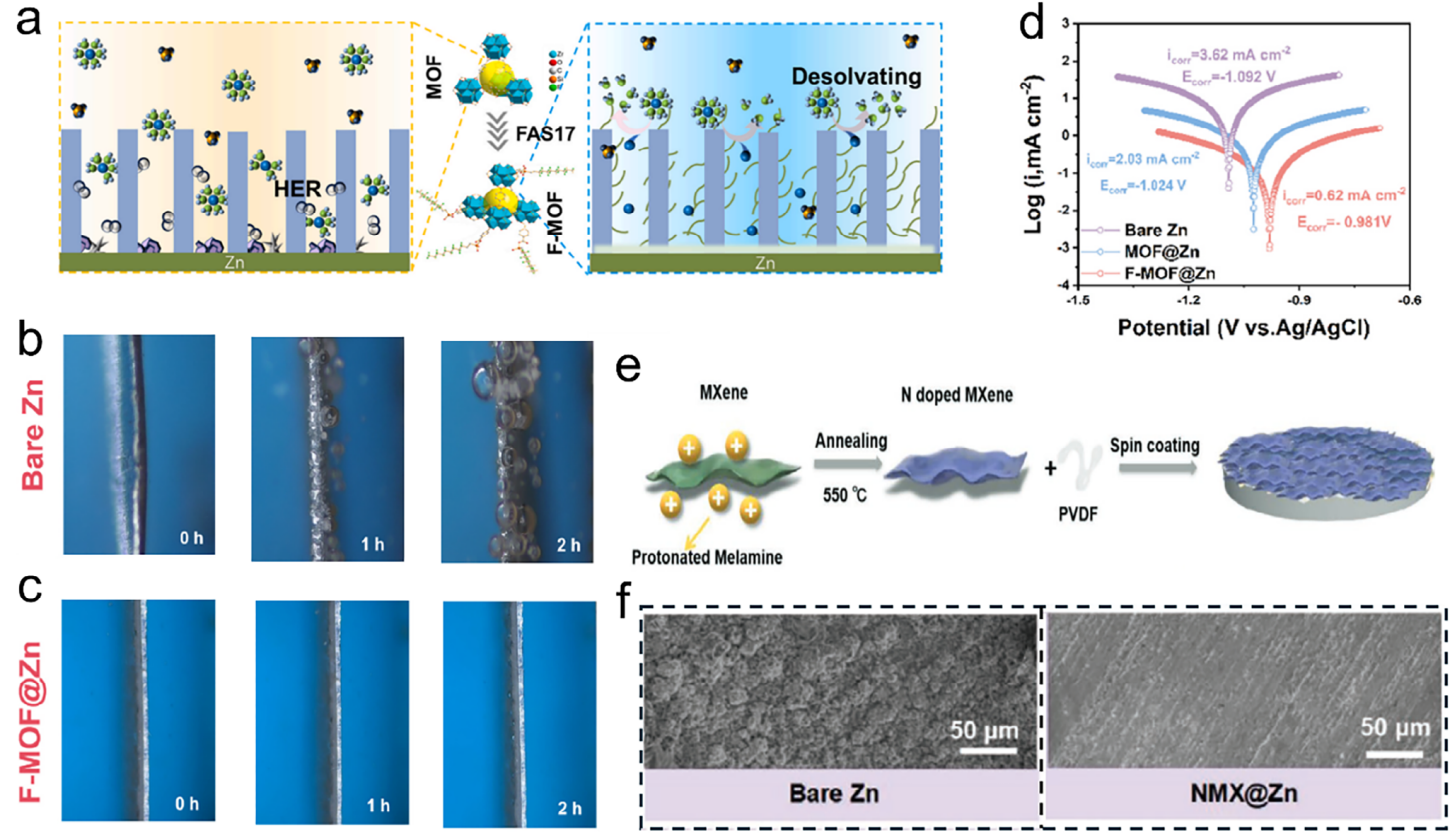
a) schematics of HER and electrochemical corrosion on MOF@Zn and F‐MOF@Zn electrode. In situ optical microscopy at 10 mA cm^−2^ b) bare Zn. c) F‐MOF@Zn d) corrosion behavior through the Tafel plot. Reproduced with permission.^[^ ^109^ ^]^ Copyright 2024, Elsevier. e) Schematic illustration of NMXene@Zn foil. f) SEM images after deposition for 1 mAh cm^−2^ of bare Zn and NMXene@Zn. Reproduced with permission.^[^ ^110^ ^]^ Copyright 2023, Wiley‐VCH.

Likewise, Wang et al. worked on a hydrophobic fluoro silane‐based metal‐organic framework (F‐MOF) to reduce HER, dendrites, and corrosion by promoting desolvation on the anode surface for even Zn deposition.^[^ ^109^ ^]^ Their work changes the MOF surface by adding fluoroalkyl silane (F‐MOF) as the synthetic (SEI) interface (SEI) to manage the desolvation and deposition process at the Zn anode. Merging a fluorodecyl‐silicon chain (‐Si(CH_2_)_2_(CF_2_)_7_(CF_3_)) with enhanced molecular elasticity and reduced surface energy developed in the formation of a hydrophobic coating on the MOF surface. In contrast, the Zn anode after MOF coating (MOF@Zn), then with F‐MOF coating (F‐MOF@Zn), not only supplied equivalent even channels for Zn^2+^ but also served equally as a hydrophobic frame which assisted as the buffer layer, splitting active Zn^2+^ from the bulk electrolyte [Zn (H_2_O)_6_]^2+^. This procedure caused a more settled complex generation with ligands in the F‐MOF channels, degraded side reactions present on the Zn anode surface, stifled HER, and reduced Zn_4_SO_4_(OH)_6_⋅xH_2_O assembling and H_2_ gas bubbles, thus upholding corrosion on the Zn anode (**Figure** **12**a). Another verification through Density Functional Theory (DFT) calculations showed that the hydrophobic changes of ‐Si(CH_2_)_2_(CF_2_)_7_(CF_3_) not only isolate water molecules from the surface of F‐MOF but also increase the desolvation of [Zn (H_2_O)_6_]^2+^, raising the local concentration. This adaptation increases channel regulation and improves interactions among channel walls by encouraging Zn^2+^ diffusion inside the F‐MOF structure. Subsequently, it allows a reversible Zn plating/stripping process on the Zn anode.

To investigate the inhibitory causes of the F‐MOF coating on Zn dendrite initiation and HER, the Zn plating process of bare Zn, MOF@Zn, and F‐MOF@Zn electrodes via in situ using optical microscopy at 10 mA cm^−2^ with the areal capacity of 5 mAh cm^−2^ is conducted. Through the initial hour, the surface of the bare Zn anode revealed disorderly Zn clusters and tiny bubbles. Later, within 2 h, Zn dendrites and H_2_ gas bubbles steadily wrapped the electrode (Figure 12b). The MOF@Zn slightly restricted dendrite growth but could not control the suppression of H_2_ bubbles formation until 2 h of Zn plating. The F‐MOF@Zn electrode appeared to be a homogenous Zn coating throughout the 2 h duration with insignificant Zn dendrites and H_2_ bubbles (Figure 12c). To assess the cause of corrosion in the electrolyte, a three‐electrode technique on bare Zn, MOF@Zn, and F‐MOF@Zn as working electrodes, platinum foil used as a counter electrode, and Ag/AgCl as the reference electrode, respectively, to test Tafel polarization curves. The monitored corrosion current density on F‐MOF@Zn is 0.62 mA cm^−2^, drastically less than on MOF@Zn at 2.03 mA cm^−2^ and bare Zn at 3.62 mA cm^−2^ (Figure 12d). The existence of F‐MOF not only distinctly lowered the corrosion current density but also altered the corrosion potential from −1.092 V on bare Zn to −1.024 V on MOF@Zn and −0.981 V on F‐MOF@Zn versus Ag/AgCl. The F‐MOF‐coated Zn anode showed a long cycling life (2000 h) at 1 mA cm^−2^ and 0.50 mAh cm^−2^, drastically exceeding the life of the bare Zn anode for 225 h, and the MOF‐coated Zn anode for 751 h. Moreover, coin and pouch cells with F‐MOF@Zn//MnO_2_ demonstrated exceptional electrochemistry, working at 91.10% capacity retention even after 1000 cycles.

Gao et al., produced an artificial layer for SEI by nitrogen‐doped MXene through a post‐annealing process in the presence of melamine.^[^ ^110^ ^]^ This highly conductive artificial layer makes a homogenous local electric field and increases Zn deposition activity. First, layered Ti_3_C_2_Tx MXene (MX) was compiled with protonated melamine via electrostatic interaction, annealing at 550 °C in the presence of N_2,_ and acquired NMX (Figure 12e). Scanning electron microscopy (SEM) confirmed the deposition morphology of the Zn anode. The NMX@Zn electrode retained a smooth surface after Zn deposition. Conversely, the bare Zn electrode showed many massive aggregates representing the irregular deposition of Zn^2+^ (Figure 12f). The long cycling stability of several electrodes was assessed through a symmetric cell. The battery with NMX@Zn electrode displayed the longest cycle time of 1900 h at 1 mA cm^−2^ and 1 mA cm^−2^, for MX is 468 h and bare Zn 395 h. Still, at an extremely high current density of 10 mA cm^−2^, the cycle life of NMX@Zn stayed stable for 1600 h, which specifies the potential of NMX for controlling dendrite formation. In summary, ASEI facilitates uniform charge distribution and better adsorption energy for Zn, which reduces the nucleation barrier and consequently accelerates the Zn^2+^ deposition kinetics. The Zn anode with ASEI is multifunctional and simple to prepare, which can ultimately advance the industrialization of ZIBs. **Table** **2** summarizes the role of constructing ASEI on the Zn anode.

**Table 2:** Role of constructing ASEI on Zn anode.

ASEI on Zn anode	Current density [mA cm^−2^]	Cycling Stability [h]	Refs.
phenyl phosphonic acid	5	1100	[111]
Sodium and calcium bentonite	0.5	1400	[112]
imidazole‐2‐carboxaldehyde (2‐ICA)	20	800	[113]
pyromellitic acid (PA)	3	3500	[114]
Sodium 4‐vinylbenzenesulfonate (VBS)	1	1800	[115]
1,4‐thioxane (TX)	20	500	[116]
Ce‐MOF‐808	3	3200	[117]
porphyrin‐based porous organic polymers	0.5	1200	[118]
sulfonated polyaniline (S‐PANI)	0.5	1500	[119]
three covalent triazine (DCPY‐CTF, CTF‐1 and FCTF)	0.25	4000	[120]
fluorinated monomer (polyacrylic acid‐2‐(Trifluoromethyl) propenoic acid	0.5	3000	[121]
polyacrylonitrile (cPAN)	0.5	600	[122]
organic‐inorganic coating layer (Nafion–TiO_2_)	0.5	1750	[123]
calcium alginate (CA) hydrogel	0.5	2200	[124]
poly(2‐vinylpyridine) (P2VP)	1	600	[125]

#### 3.2.2 SEI Through Electrolyte Additive

**Figure 13:**
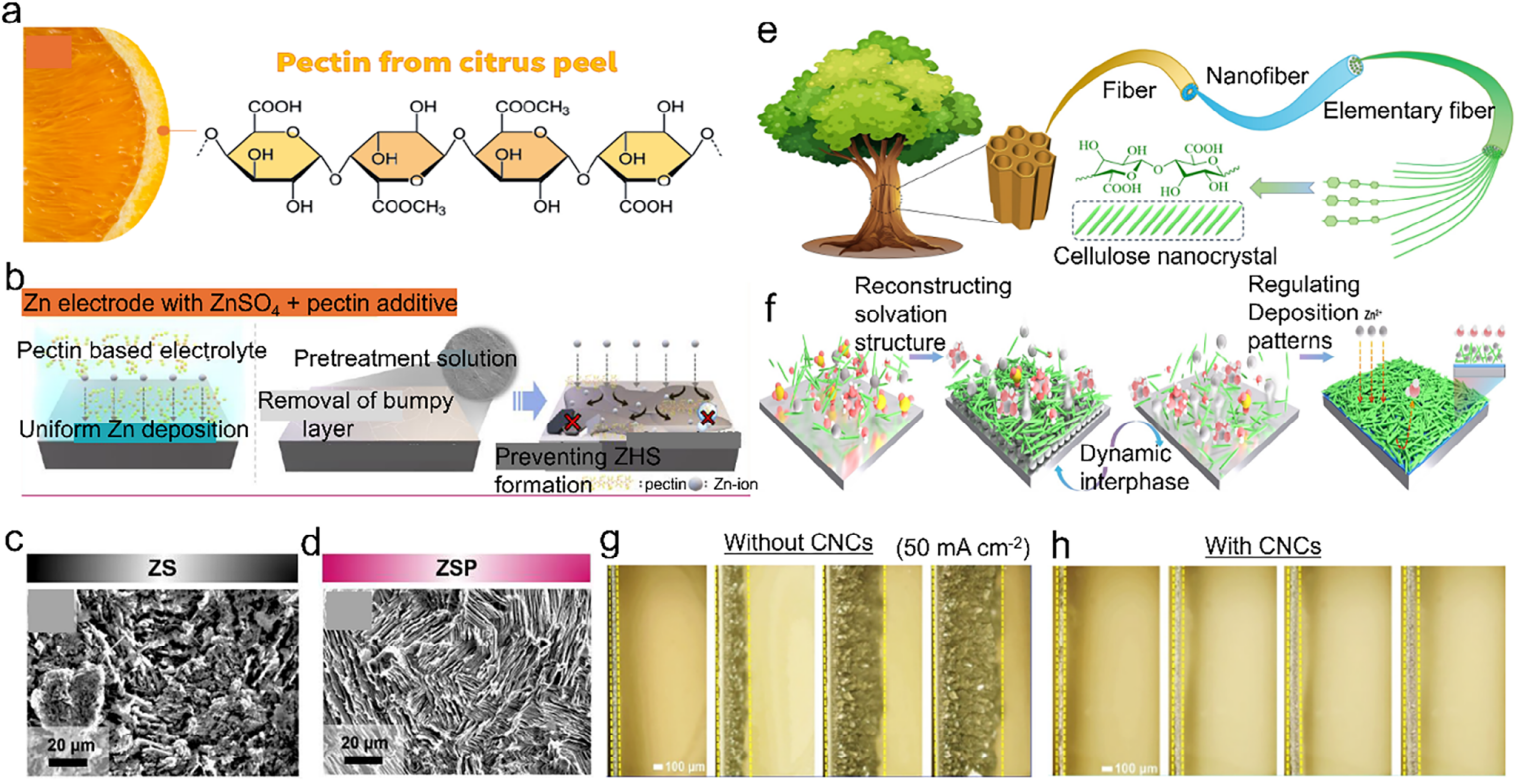
a) Schematic illustration of pectin. Schematics of acidic pectin‐based ZSP electrolyte on Zn electrode. b) SEM images of Zn electrode after stripping c) ZS(Zn‐sulfate) d) ZSP (Zn‐sulfate pectin) electrolyte. Reproduced with permission.^[^ ^126^ ^]^ Copyright 2024, Wiley‐VCH. e) Schematics for fabricating CNCs from wood. f) deposition of Zn on metallic Zn in the presence of a CNCs electrolyte additive g) images of Zn deposition through in situ Optical microscopy at 50 mA cm^−2^ and 50 mAh cm^−2^. Reproduced with permission.^[^ ^130^ ^]^ Copyright 2024, Wiley‐VCH.

SEI is usually developed by adding suitable compounds to the electrolyte to proceed with an in situ formation of SEI during cycling to enhance the performance of ZIBs. The basic purpose of additives is to develop a protective layer at the electrode and electrolyte interface by regulating the distribution of Zn^2+^ to avoid unwanted by‐products and side reactions.^[^ ^118^ ^]^ Utilizing additives in the electrolyte is an innovative way of tuning the composition of the SEI on the Zn anode surface during the battery operation to achieve longer cyclic life. Jang et al. used complex acidic polysaccharide (pectin), which is generally found in the inner walls of fruits and used to bind water as a gel or thickening agent.^[^ ^126^, ^127^ ^]^ Pectin is naturally filled with D‐galacturonic acid, a hydroxyl group (‐OH), aldehyde group (‐CHO), and carboxylic group (‐COOH), which ionize in H_2_O and relatively reduce the pH value of aqueous solutions (**Figure** **13**a).^[^ ^128^ ^]^ The impacts of pectin can be reviewed as follows: the irregular Zn surface with ZS electrolyte not only speeds up the side reactions, such as Zinc hydroxide sulfate (ZHS) growth and HER production but also allows Zn dendrites to grow because of inhomogeneous Zn^2+^ flux. On the contrary, the acidic electrolyte using pectin as an additive restrains the side reactions and generates homogeneous deposition, thus improving the reversibility of the ZIBs.

A pectin‐based mixture reveals an even surface by eliminating the irregular layer on the Zn foil (Figure 13b). The altered Zn electrode in the pectin mixture helps hinder the ZHS and decrease the overpotentials. In the ZIB working operation, the first process involves a discharging and stripping reaction on the Zn electrode. After the first stripping process, if the morphology of the Zn surface reveals an inhomogeneous and rough structure, the formation of dendrites speeds up.^[^ ^129^ ^]^ Hence, the stripped Zn anode intensely affects the successive charging process. The asymmetric (Zn||Cu) discharged at 2 mA cm^−2^ to examine the stripping performance on the Zn surface with electrolytes. After the first stripping process, SEM images of the Zn anode in the ZS electrolyte showed an irregular and bumpy morphology with complicated tiny particles (Figure 13c). In comparison, the Zn electrode in the presence of Zinc sulfate with pectin (ZSP) exhibited an efficiently stripped morphology (Figure 13d). Consequently, the ZSP electrolyte helps stop the growth of ZHS and restrain HER, thus improving the reversibility of the ZIBs. The Zn//Cu arrangement with ZSP demonstrated a longer cycle for more than 23 times elongated than without the ZSP cell, including ZS at 1 mA cm−2 with a 1 mAh cm−2 deposition capacity. Wu et al. worked on cellulose nanocrystals (CNCs) as an electrolyte additive, which enhanced Zn‐ion transportation and improved the coordination of the electrolyte/electrode interface.^[^ ^130^ ^]^ CNCs are bio‐based, environmentally friendly materials manufactured for industries in large quantities per day worldwide for numerous fields owing to their exceptional physical and chemical properties, for example, higher aspect ratio, higher thermal stability, and plentiful surface functional groups. The carboxylated and hydroxylated CNCs were obtained via oxidation technique from cellulose (Figure 13e).

Being high in oxygen functional groups the CNC assists in rebuilding the solvation structure by driving down the participation of Zn^2+^ with water molecules and boosting the kinetics of Zn^2+^ in the electrolyte. Moreover, the intrinsic electronegativity of CNCs and strong adsorption interaction with Zn atoms permit migration in the presence of an electric field by homogenization and acceleration of the Zn^2+^. Surprisingly, CNCs allow the growth of an effective interface that suggests stable protection by supporting speedy but effective Zn plating and stripping performance, regulating the ion and electric field distribution, and granting a physical and chemical buffer for the Zn anode (Figure 13f). In situ microscopy is done to analyze the zinc deposition manner in CNC additives. At a super high current density of 50 mA cm^−2^, irregularly accumulated chunks arose instantly after 30 s in the ZnSO_4_ electrolyte, commanding the evolution of critical dendrites on the Zn anode (Figure 13g). On the contrary, the deposition of Zn in CNCs additives was consistent, and it was even verified that constant Zn^2+^ flow and electric field were produced (Figure 13h). Also, the Zn//MnO_2_ full cells with CNCs additive exhibited higher cyclic stability with an incredible capacity retention of 76.30% and CE of ≈100% even after 3000 cycles at 10 A g^−1^. Considering the above improvements in electrolyte additives, Naveed et al., introduced the amino acid D‐Phenylalanine (DPA) in 1 M ZnSO_4_ electrolyte as a bifunctional electrolyte additive.^[^ ^131^ ^]^ The DPA molecules recommend excellent and ideal adsorption with the Zn anode via a charged carboxyl group controlling the Zn plating and assembling an in situ SEI on the electrode surface, supporting fast Zn^2+^ transport kinetics. The electrolyte with DPA‐additive executed outstanding electrochemical stability of Zn anode for over 3000 h at (0.25 mA cm^−2^, 0.125 mAh cm^−2^), 114 times better than without DPA electrolyte.

**Figure 14:**
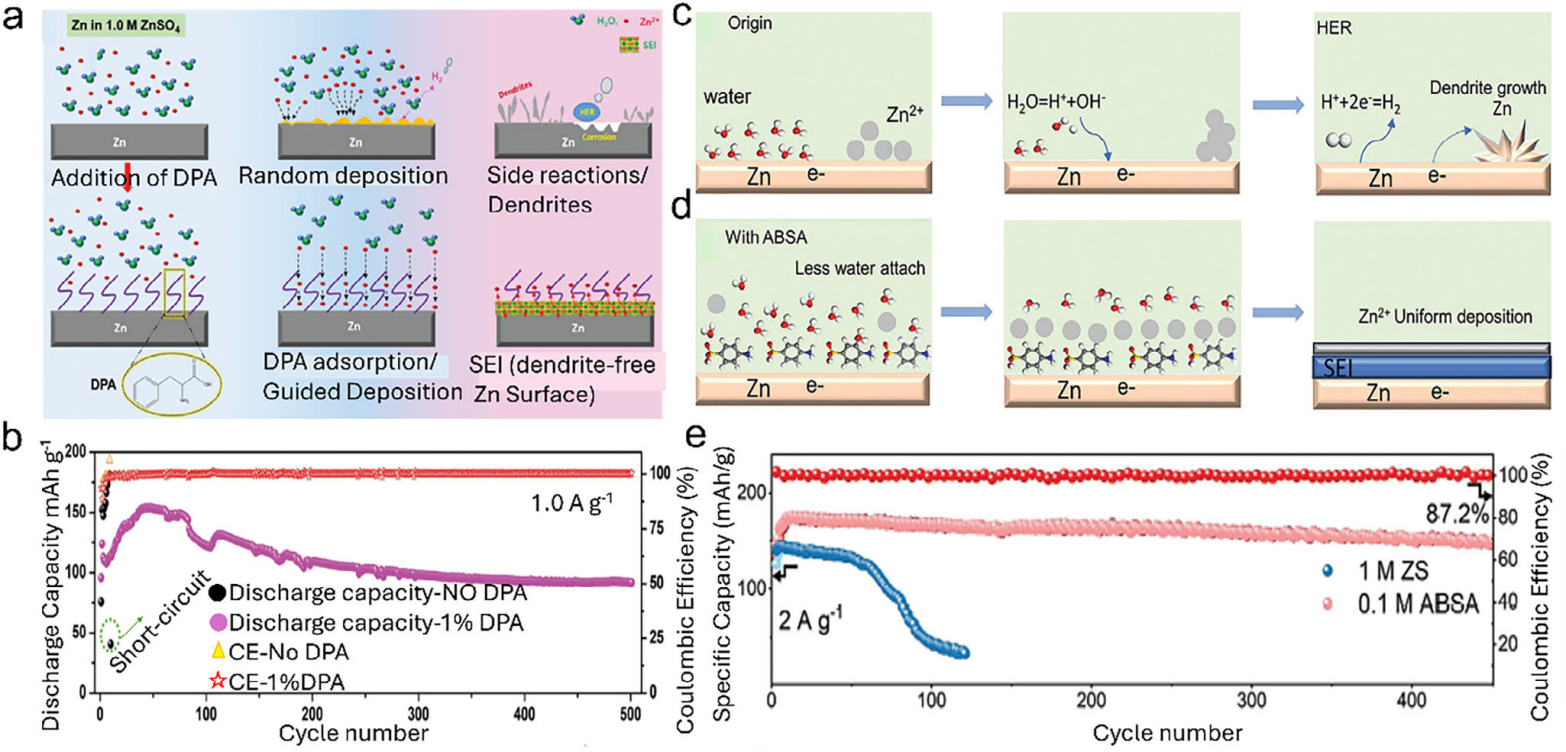
a) Schematic showing Zn deposition with and without additive, b) cycling performance of Zn//MnO_2_ full cell with and without electrolyte additive. Reproduced with permission.^[^ ^131^ ^]^ Copyright 2024, Wiley‐VCH. Schematics of the electrolyte and electrode interface c) without additive d) with additive e) cycling stability of 1 molar ZnSO_4_ and 0.1 M additive (ABSA) at 2 A g^−1^. Reproduced with permission.^[^ ^35^ ^]^ Copyright 2024, Wiley‐VCH.

The illustration of Zn plating with and without the DPA additive in **Figure** **14**a explains how the preferential concentration of DPA molecules on the zinc surface assisted the patterned flow of Zn^2+^ in the direction of the electrode surface, leading to smooth plating. The electrolyte additive can construct in situ SEI on the Zn anode interface. A trace amount of electrolyte additive is expected to adsorb and cooperate with the electrode surface to produce the SEI. Moreover, the reduced polarization of Zn/MnO_2_ full cell with a DPA additive proposed quicker kinetics. The cycling performance of the full cell with and without the DPA additive was investigated at 1 A g^−1^ with galvanostatic charging/discharging (Figure 14b). The DPA additive cycled for over 500 cycles, exhibiting outstanding cycling performance with competent capacity retention, whereas, without additive, it underwent an immediate short circuit, indicating battery failure. Li et al. proposed 4‐aminobenzenesulfonic acid (ABSA) as an additive into the regular ZnSO_4_ electrolyte due to which an intermolecular network occurred during the hydrogen bonding interactions involving the ─SO_3_H and─NH_2_ groups in ABSA, determining a settled and efficient pre‐adsorption layer of ABSA on the Zn anode.^[^ ^35^ ^]^ The pre‐adsorbed ABSA layer may lower the excessive contact between the metal Zn anode and the water molecules, causing an incredible inhibition in the corrosion and side reactions like HER of the metal zinc electrode (Figure 14c,d). The contrast of the settings with and without ABSA additive performed the valuable effect of the ABSA pre‐adsorption layer for controlling the side reaction and commanding the dense deposition of Zn^2+^ on the Zn metal anode. Furthermore, 0.10 M ABSA electrolytes demonstrated excellent stability during long cycling for 450 cycles at 2 A. This signifies a considerable development in cycling performances compared to electrolytes without additives (Figure 14e). Moreover, the full cell with the VO_2_ cathode and high mass loading of 10 mg cm^−2^ was explored to expand the utilization of the zinc anode.

Consequently, these experimental results have exposed 10 000 mAh cm^−2^ accomplishment in accumulating plating/stripping ion capacity. The Zn^2+^ intercalation confirmed the cyclic stability of the battery. Similarly, Ma et al. introduced 3,5‐bis(trifluoromethyl)pyrazole (TEMP) in various alkaline, acidic, and non‐aqueous electrolytes.^[^ ^132^ ^]^ TEMP showed excellent performance in all the classes of electrolytes and, above all, in the weak acidic aqueous electrolyte. **Table** **3** summarizes the role of various electrolyte additives and their impact on full cells in ZIBs.

**Table 3:** Role of various electrolyte additives and impact on the full cell in ZIBs.

Electrolyte	Additive	Additive function	Current density	Capacity	Cathode	Cycle number	Refs.
1 M ZnSO_4_	Tripropylene glycol (TG)	Regulating overpotential	4 A g^−1^	124.48 mAh g^−1^	MnO_2_	1000	[133]
ZnCl_2_	Hydroxyl‐rich (α‐D‐glucose)	Fast Zn^2+^ desolvation	2 A g^−1^	38.7 mAh g^−1^	Polyaniline (PANI)	10000	[134]
2 M ZnSO_4_	Triethyl phosphate (TEP)	Weakening of Zn^2+^ solvation	3 A g^−1^	116 mAh g^−1^	LiFePO_4_	1500	[135]
2 M ZnSO_4_	(1,2‐Ethanedisulfonic acid (EDA))	Build Strong adsorption energy with Zn anode	5A g^−1^	1 mAh cm^−2^	NH_4_V_4_O_10_	2000	[136]
2 M ZnSO_4_	sodium dodecyl benzene sulfonate (SDBS)	Optimization of Zn^2+^ solvation sheath	0.5 A g^−1^	0.2 mAh cm^−2^	LiFePO_4_	500	[137]
2 M ZnSO_4_	disodium succinate (SADS)	Regulate the solvation structure of Zn^2+^	5 A g^−1^	47 mAh g^−1^	I_2_	3000	[138]
2 M ZnSO_4_	dipeptide glycylglycine (Gly‐Gly)	The interfacial layer isolates the electrode from water molecules	200 mA g^−1^	206.5 mAh g^−1^	MnO_2_	500	[139]
2 M ZnSO_4_	ZIF‐8	Uniform ion flux	1 A g^−1^	106.1 mAh g^−1^	MnO_2_	500	[140]
1 M ZnSO_4_	trisodium methyl glycine diacetate (Na_3_ MGDA)	Developed a hydrophobic electrical double layer (EDL)	3 A g^−1^	30.25 mAh g^−1^	V_6_O_13_	1300	[141]
ZnSO_4_	dodecyl trimethyl ammonium chloride (DTAC)	Elevates nucleation overpotential	5 A g^−1^	149.44 mAh g^−1^	MnO_2_	2000	[142]
ZnSO_4_	nicotinic acid (NA)	Tunned solvation	2 A g^−1^	1 mAh cm^−2^	activated carbon (AC)	700	[143]
ZnSO_4_	N‐Acetyl‐ϵ caprolactam (N‐ac)	Reduce H_2_O within Zn^2+^ solvation sheath	2 mA cm^−2^	1 mAh cm^−2^	ZnV_6_O_16_	2000	[144]
1 M ZnSO_4_	N‐allylthiourea (ATU)	Uniform Zn deposition achieved	200 mA g^−1^	150 mAh g^−1^	VO_2_	300	[145]
ZnSO_4_	4‐hydroxybenzoic acid sodium salt (PHB)	Increase the steric effect	10 A g^−1^	80.50 mAh g^−1^	NH_4_V_4_O_10_	1000	[146]
ZnSO_4_	sodium citrate (SC)	Solvation shell regulation	1 A g^−1^	100.80 mAh g^−1^	NaV_3_O_8_·1.5 H_2_O (NVO)	400	[147]

#### 3.2.3 Eutectic Electrolyte

**Figure 15:**
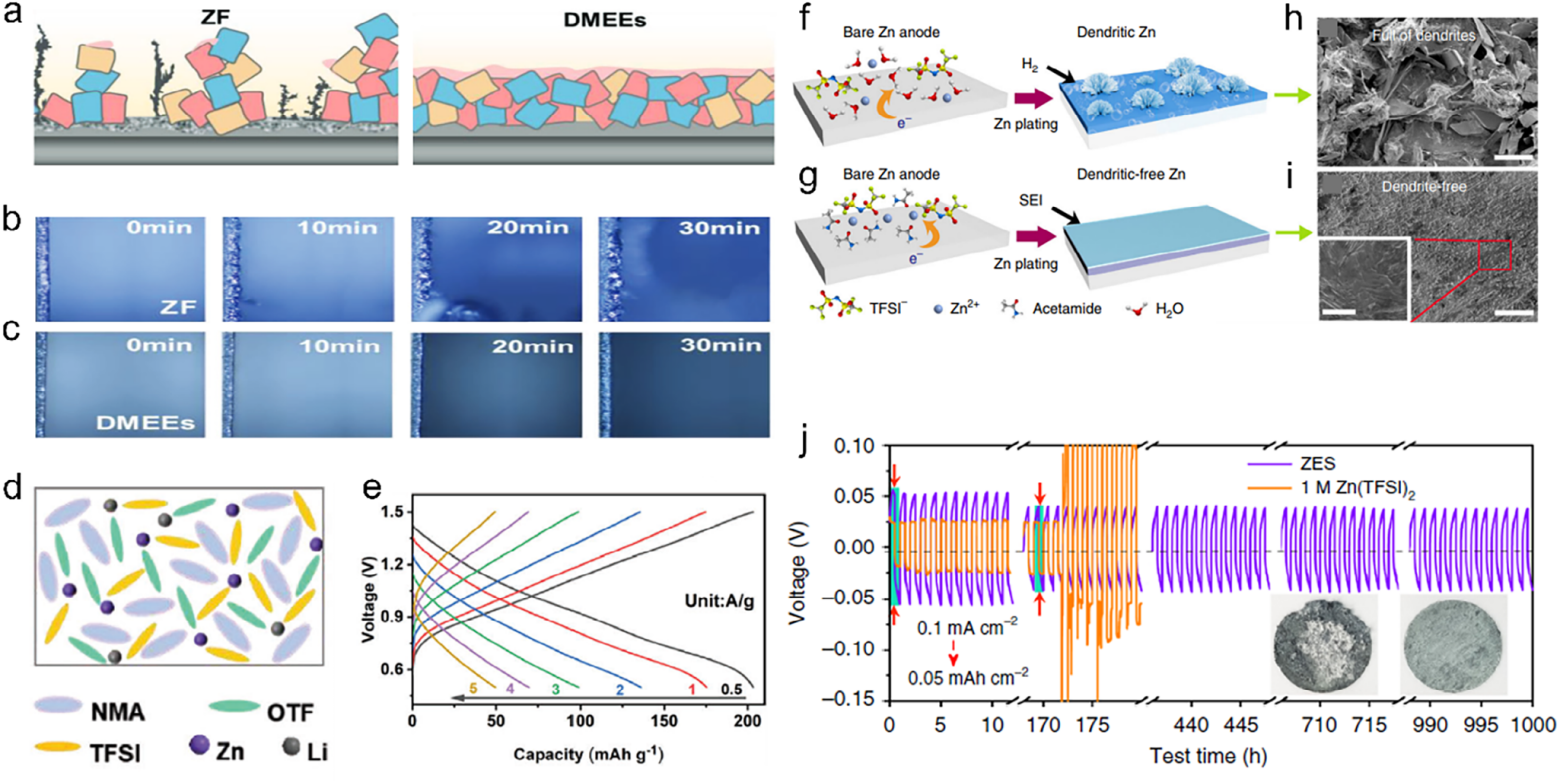
a) Schematics of dendrite formation and side reactions in the presence of ZF and with SEI layer of DMEEs on Zn anode. In situ optical microscopy b) without DMEEs c) With DMEEs d) solvation of Zn^2+^ and Li^+^ by ion aggregation in DMEEs and NMA molecules e) charge and discharge voltage curve of Zn//PANI full with DMEEs. Reproduced with permission.^[^ ^153^ ^]^ Copyright 2024, Wiley‐VCH. f) Formation of Zn dendrite and HER in 1 M Zn (TFSI)_2_, g) uniform Zn deposition in the presence of ZES. SEM images showing Zn deposition h) in 1 M Zn(TFSI)_2_ i) in the presence of ZES electrolyte at 1 mA cm^−2^ and 0.5 mAh cm^−2^ j) voltage response for Zn symmetric cell in ZES and 1 M Zn(TFSI)_2_ at 0.1 mA cm^−2^ and 0.05 mAh cm^−2^ for 1000 cycles (optical images after 180 cycles).Reprinted with permission.^[^ ^154^ ^]^ Copyright 2019, Springer Nature.

The eutectic electrolytes present an interesting choice for use in battery technology because of their unique physicochemical properties, such as high ionic conductivity, nonflammability, low viscosity, and non‐volatility. These types of electrolytes can be made with a combination of a range of chemicals such as choline chloride, urea, ethylene glycol, acetamide, etc., with a metal salt, e.g., AlCl_3_, FeCl_3_, ZnCl_2_, etc, which can be broadly used in zinc‐metal ion batteries because of its magnificient electrochemical and thermal stability. The scheme of eutectic electrolyte was initially given in 2003 by Abbott et al, whereas recent electrolyte modifications include water‐in‐salt electrolyte (WISEs), deep eutectic solvents (DES), electrolyte optimization, and hydrogel, etc.^[^ ^148^ ^]^ For example, Liu et al. proposed a ternary eutectic electrolyte using Zinc perchlorate hexahydrate, LiCl, and butanedinitrile (BD) to investigate a temperature range from lower (−20 °C) to higher (70 °C) and achieved excellent stability of the full cell for above 1000 cycles.^[^ ^149^ ^]^ Similarly, Jiang et al., introduced anti‐freezing deep eutectic solvents using Zn(ClO_4_)_2_ + Mg(ClO_4_)_2_ and made a chemical environment with a low freezing point at −116.92 °C.^[^ ^150^ ^]^Moreover, Tang et al. used choline chloride, ZnCl_2_, and urea to make an anhydrous deep eutectic electrolyte and obtained extraordinary results, 11 500 h cyclic stability for a symmetric cell.^[^ ^151^ ^]^ Further, extending to this idea, Jiang et al. reported a ternary eutectic aqueous electrolyte system by adding *N*‐ethylacetamide (Nea), Zn(OTf)_2_, and water. The basic idea was to develop an electric field by desiccating the protective layer and removing the side reactions. The cycle life of the full cell exceeds for over 5000 with 100% Coulombic efficiency at 1.0 A g^−1^ and 2.0 A g^−1^.^[^ ^152^ ^]^ Depending on the unique qualities of eutectic electrolytes, Li et al. introduced a salt‐based strategy by designing a dual metal salt‐derived ternary eutectic electrolyte (DMEES) to inhibit the reoccurrence of dendrites.^[^ ^153^ ^]^ DMEES was designed by mixing zinc trifluoromethane sulfonate Zn(OTF)_2_, *bis*(trifluoromethanesulfonyl)imide, and a neutral ligand of *N*‐methyl acetamide (NMA). The NMA is a beneficial ligand for DEEs because its dipolar character appears from two polar functional groups of ─NH and C═O, which supports the efficient coordination among cations and anions of the metal salt. Whereas the other amide ligands (such as thiourea, urea, and acetamide) introduce a methyl group on the amino in NMA disturbs intramolecular hydrogen bond interactions, causing minimal viscosity of the formulated DEEs. The DMEES is rich in anion Zn‐ion solvation and makes an inorganic hybrid SEI, effectively inhibiting dendrites on the Zn anode. The neutral ligands and salts are mixed by maintaining the homogeneity of the liquid. The DMEEs exposed interesting chemical properties compared to the Zn(OTF)_2_ aqueous electrolyte (ZF). Moving on, ZF endured severe corrosion and dendrite progression, and the produced complexes, such as ZnS and ZnF_2_, independently dispersed on the Zn anode. An inorganic‐hybrid SEI having ZnSO_3_, ZnCO_3_, ZnF_2_, ZnS, ZnO, and organic components fabricated on DMEEs because of the decomposition of anions and NMA generated by the dual‐anion solvation structure of DMEEs, which restrained side reactions and functioned the uniform Zn^2+^ deposition to attain vastly reversible Zn anode (**Figure** **15**a).

In situ optical microscopy analyzed the Zn^2+^ deposition at 1 mA cm^−2^ for 30 min. The Zn^2+^ preferentially deposited on the initially bulging tips and continued to grow in the same position associated with the evolution of H_2_ gas bubbles in ZF, indicating severe dendrite growth and HER (Figure 15b). Comparatively, the deposition of Zn in DMEEs provided a compact and regular morphology without H_2_ gas production even after 30 min. Additionally, the efficiency of DMEEs in regulating Zn^2+^ deposition also organizes great reversibility of the Zn anode (Figure 15c). In the meantime, the intense cation‐dipole interactions among C═O and metal cations led to the weak hydrogen bond interactions in NMA molecules and the insertion of metal salt through NMA molecules, which participated in the dissociation of metal salt and produced more ion pairs and ion agglomerate and delocalizing the charge of both (cation, anion) by producing smooth and constant DMEEs (Figure 15d). To explore the charge/discharge curve at various current densities in (Figure 15e), at 0.5 A g^−1^, the capacity 195.50 mAh g^−1^ depicted quick interface reaction kinetic. Similarly, Qiu et al. introduced another eutectic electrolyte based on acetamide‐Zn(TFSI)_2_, where acetamide acts as a potential solvent candidate.^[^ ^154^ ^]^ The Zn anode is protected by exploring the in situ formation of ZnF_2_. The ZnF_2_‐rich ionically permeable SEI layer and stabilized Zn electrochemistry have shown 100% CE. Mixing Zn (TSI) and acetamide, known as zinc eutectic savant (ZES), used several percentages of molar ratios. Using ZESs and the potential in situ SEI protective layer can greatly impact the Zn deposition. With a deposition capacity of 0.50 mAh cm^−2^, a weak structure with unrestrained formation of Zn dendrites emerged in 1 M Zn (TFSI)_2_, which is certainly considered for the low CE (Figure 15f). Whereas, for ZES electrolytes, homogenous and dendrite‐free Zn deposits at a higher capacity of 2.50 mAh cm^−2^ can be seen in (Figure 15g).

Explore surface morphology, SEM images showed the presence of dendrites without ZES (Figure 15h). Thus, it is logical to suppose that this added Zn‐electrolyte interface controls the reversible Zn/Zn^2+^ redox reaction with great Zn^2+^ movement and deposition (Figure 15i). The excellent performance of ZES for protecting the Zn anode is determined through galvanostatic conditions in the Zn//Zn symmetric cell. Despite the large polarization, batteries using ZES exposed viable electrochemical performance in comparison to those with 1 M Zn (TFSI)_2_. As observed, the overpotential steadily decreased from 55 to 39 mV when cycled at 0.1 mA cm^−2^ in the presence of ZES (Figure 15j). As the current density increased to 0.50 mA cm^−2^, the same cell with ZES continued to function gradually for an additional 1000 h. The surface morphology of Zn after 180 cycles in ZES is uniform (inset right); however, Zn protuberances are visible in ZF (inset left). In summary, eutectic electrolytes present an interesting choice of electrolytes to stabilize the Zn anode for ZIBs. However, their utilization is still in the early stages and requires extensive investigations.

## 4 Advanced Characterization Techniques

It is particularly crucial to understand the complex electrochemical processes and enhancing their performance of ZIBs. These techniques, including in situ and operando methods, provide insights into the materials' behavior during pre‐post battery operation, which is essential for optimizing their design and functionality. While these advanced techniques significantly contribute to the understanding and improvement of ZIBs challenges, such as dendritic growth, side reactions remain prevalent. Addressing these issues is essential for the practical application of ZIBs in large‐scale energy storage solutions. Here, first we will discuss the Electrochemical Quartz Crystal Microbalance (EQCM) based observation, then for elemental analysis we will explain some studies based on XPS, and for morphological study, we will give a soft touch to various advanced microscopic techniques, for example, AFM, SEM, and TEM. Further, spectroscopic analysis of additives in the electrolytes will be studied through in situ XRD, Raman, SAXS, XCT, FTIR, and TOF‐SIMS.

### 4.1 Electrochemical Quartz Crystal Microbalance (EQCM)

Measures of mass changes of the electrode during cycling are very useful for studying Zn deposition/dissolution behavior in ZIBs. Therefore, an electrochemical quartz crystal microbalance (EQCM) technique is used because this technique plays a crucial role in ZIB research by providing insights into the electrochemical processes and degradation mechanisms. These techniques enable real‐time monitoring of mass changes and interfacial phenomena, which are essential for understanding battery performance and longevity. Betts et al. studied the in situ EQCM technique to monitor for the observation of mass variations at the electrode interface during charge/discharge cycles, revealing the formation of SEI layers and their impact on cycling stability.^[^ ^155^ ^]^ The EQCM‐D results for 1 M aqueous ZnSO_4_ show that with each of the five cycles of CV, additional mass accumulates at the electrode. This occurs in two potentially dependent stages. The initial mass increase occurs at ≈0.40 V, corresponding with the significant increase in SO_4_ peak intensities detected in the IR spectra. The adsorbed layer likely comprises solvated cations represented as adsorbed Zn_4_SO_4_(OH)_6_·xH_2_O. At ≈0 V, a subsequent increase in mass is detected at the initiation of zinc reduction, during which Zn can adsorb directly onto the electrode surface. As the cycle advances, mass desorbs from the surface but does not completely revert to its adsorption value, signifying the development of a Zn interphase layer. The formation of Zn_4_SO_4_(OH)_6_·xH_2_O in a slightly acidic ZnSO_4_ electrolyte, nevertheless, its loose and porous characteristics render it ineffective as a passivation layer, leading to ongoing corrosion of deposited zinc and subsequent battery failure.^[^ ^156^ ^]^

**Figure 16:**
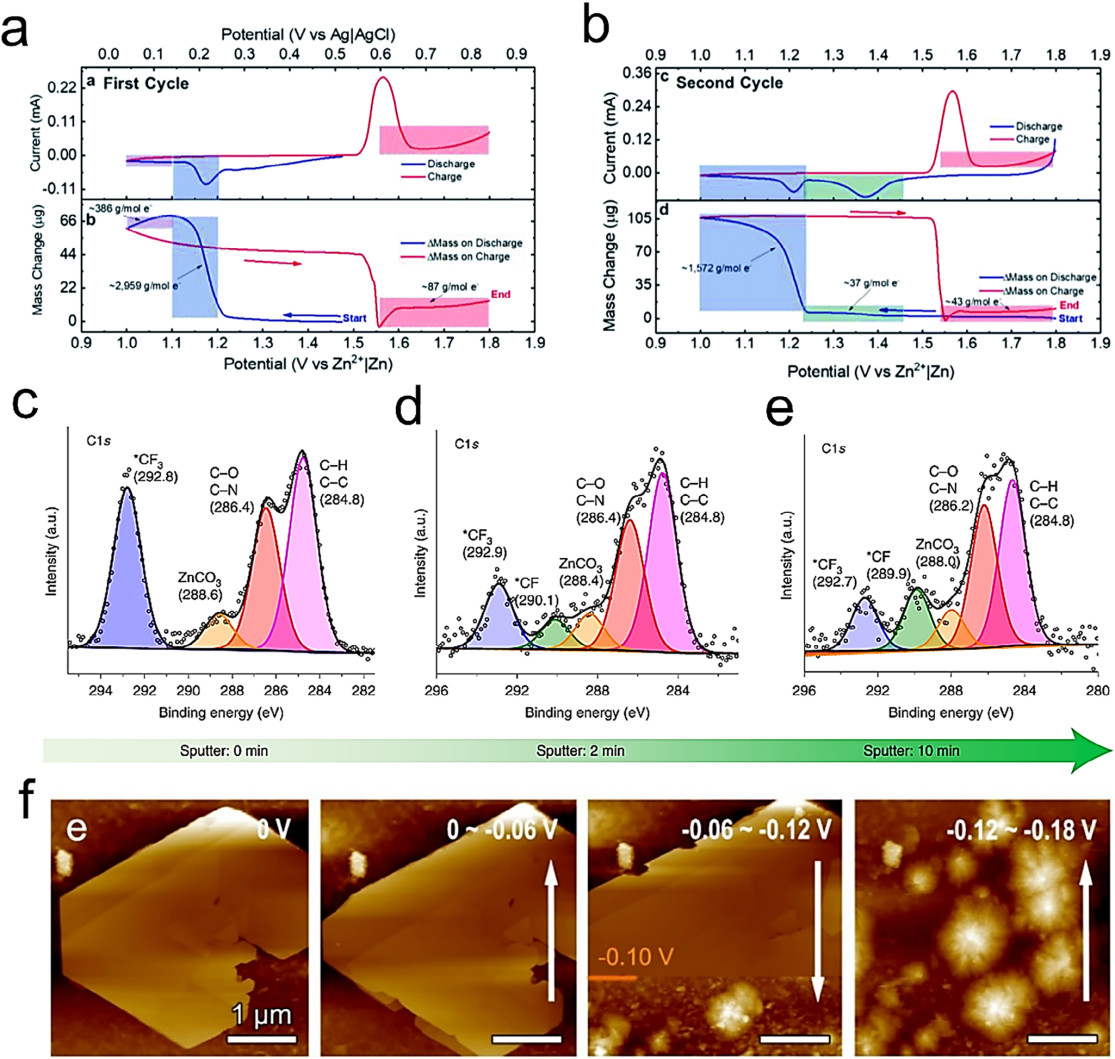
a) 1st cycle CV and b) EQCM of MnO_2_/Au electrode 1 M ZnSO_4_ + 0.1 M MnSO_4_. Reproduced with permission.^[^ ^158^ ^]^ Copyright 2021, The Royal Society of Chemistry. c) Depth profiles of F1*s* generated after Ar^+^ sputtering for 0 min d), 2 min e), and 10 min. Reproduced with permission.^[^ ^171^ ^]^ Copyright 2021, Nature Publishing Group. f) In situ potential‐dependent AFM topography images. Reproduced with permission.^[^ ^172^ ^]^ Copyright 2020, American Chemical Society.

By coupling EQCM with other techniques, such as UV‐Vis spectroscopy, researchers can track the evolution of mass and chemical composition, elucidating the effects of additives like acetate on battery performance. He et al. utilize these complimentary techniques that concurrently monitor the evolution of mass and composition.^[^ ^157^ ^]^ The synthesis and dissolution of zinc hydroxide sulfate (ZHS) and manganese oxides demonstrate the influence of acetate ions on zinc‐manganese batteries (ZnMnBs) from a different viewpoint. The concentration of acetate and the pH level significantly influence the capacity and CE of the MnO_2_ electrode; therefore, they must be tuned when developing full ZnMnBs with high‐rate capability and reversibility. EQCM has been instrumental in studying redox processes and the dissolution/deposition of zinc compounds, providing a comprehensive understanding of the electrochemical reactions occurring in ZIBs. Rodríguez‐Pérez et al. studied the redox pairs occurring during operation and are mechanistically analyzed by using a combination of structural and electrochemical approaches, highlighting the significant effect of the electrolyte addition (0.1 M MnSO_4_) in a 1 M ZnSO_4_ electrolyte.^[^ ^158^ ^]^ An EQCM has been utilized to elucidate the impact of zinc hydroxy sulfate salt (Zn_4_SO_4_(OH)_6_·nH_2_O) and zinc manganese oxide (Zn_x_Mn_y_O_z_) dissolution/deposition, which are considered significant constituents during discharge and charge states. These discoveries offer insights that are presently unavailable, facilitating a comprehensive understanding of the electrochemical reaction pathways during battery operation. The CV of an Au QCM electrode after the electrochemical deposition of MnO_2_ (MnO_2_/Au in a 1 M ZnSO_4_ + 0.1 M MnSO_4_ electrolyte) is shown in **Figure** **16**a. The morphology of the CV curve closely resembles recent investigations of MnO_2_ in zinc sulfate electrolytes. The EQCM measurements were conducted using potentials relative to Zn/Zn^2+^ rather than Ag/AgCl (Figure 16b). Despite the advantages of EQCM, the next‐generation EQCM's needs to overcome the challenges such as investigations of microporous electrode materials, which will provide critical information for pore evolution during charge/discharge.

### 4.2 In Situ X‐Ray Photoelectron Spectroscopy (XPS)

The kinetic energy of photoelectrons generated by X‐rays is measured using XPS to analyze the composition of solid materials' outermost surfaces.^[^ ^159^ ^]^ XPS can identify surface atom elemental species and chemical states at depths greater than 10 nm when combined with ion etching.^[^ ^160^ ^]^ Zinc deposits preferentially at locations with low nucleation barriers, causing dendrite growth as zinc accumulates at irregular active sites during cycles.^[^ ^161^ ^]^ Electrolyte additive silicon nanoparticles regulated early nucleation.^[^ ^162^ ^]^ The XPS spectra show peaks at 533.90 and 101.80 eV for O1*s* and Si2*p*, respectively, as well as a drop in binding energy for Zn2*p*, suggesting a novel Si─O─Zn bond after 30 s of plating. Silicon nanoparticles may help uniformly deposit zinc ions by acting as deposition sites and creating chemical interactions with the anode and zinc ions. Dendritic development is inhibited by nucleation regulation in the early phase but not during substantial deposition or protracted cycles. AILs are stable and can extend battery life for long‐term protection. The tri‐layer structure of In@Zn@In showed a stable interface, as XPS signals for Zn, In, and O elements persisted after 2000 cycles in whole cells. AILs like In@Zn@In can minimize dendrite development, however, zinc deposition volumetric variations limit their stability and sustainability after continuous cycling. Thus, in situ SEI with self‐healing capabilities are attracting interest.^[^ ^163^ ^]^ Organic electrolytes like vinylene carbonate and ethylene glycol break down to form an SEI during plating and stripping. Because of the expense and flammability concerns, SEI‐induced additions are perhaps more inclined to organic solvents.^[^ ^164^ ^]^ Elemental composition and surface valence states are verified by XPS, proving SEI production. Cao et al. created in situ hydrophobic and fluorinated surfaces using (CH_3_)_3_(CH_3_CH_2_)NOTF as an adjuvant.^[^ ^165^ ^]^ The XPS signal at 684.70 eV for ZnF_2_ confirms a fluorinated interphase layer. In addition, C1*s* valence bond analysis revealed a rich ZnCO_3_ interphase layer under ZnF_2_ (Figure 16c–e). In situ‐generated SEIs have stronger zinc ion conductivity and hydrophobicity, minimizing dendrite formation and side reactions.

The XPS can identify elements and determine their relative elemental composition at the interface, enabling complex IPL component analysis. A combined layer of inorganic and organic materials was suggested to solve the disadvantages of the inorganic layer (which fails so that water doesn't get in) and the organic layer (which has poor ion mobility). For a mixed ionic polymer layer, triethyl phosphate (TEP) was used as an electrolyte.^[^ ^166^ ^]^ XPS research reveals that the IPL surface is predominantly made up of organic compounds from TEP and OTF breakdown, as identified by Ar^+^ etching at different points along the length of the body. As etching depth increases, so does inorganic content, so Zn_3_(PO_4_)_2_ increases from 27.16% to 74.79%, ZnF_2_ from 58.42% to 71.73%, and ZnS from 12.40% to 29.86%, indicating the creation of a composite AIL with an organic exterior and inorganic contents. Despite several advantages, the XPS has the limitation of depth profiling which limits its applicability.

### 4.3 Morphological Observation

The observation of morphology in ZIBs plays a crucial role in understanding the relationship between structural changes and electrochemical performance degradation. To observe material surface on the nanoscale through AFM, SEM, and TEM is important and explained in this section.

#### 4.3.1 Atomic Force Microscope (AFM)

AFM detects interatomic forces to study interfacial nucleation and dendritic growth. In a mild acidic electrolyte (1 m ZnSO_4_), Zinc ions form spherical crystals by nucleating from a sheet‐like structure, showing anisotropic development with deposition time. In situ AFM images show the association between loose nucleation and side reactions. Due to their cost‐effectiveness and functionality, electrolyte additives have been widely used to regulate nucleation. Electrolytes might include EGME, according to Liu et al. as the AFM revealed that EGME's inherent zincophilicity allows it to adsorb onto the anode and modify interfacial deposition kinetics, smoothing the surface.^[^ ^167^ ^]^ Besides studying early nucleation, AFM analyses anode deposition morphology by showing deposition morphology more intuitively due to 3D imaging.^[^ ^168^ ^]^ They used a densified polyacrylonitrile/silicon dioxide (PAN‐SiO_2_) nanofiber to enhance solid‐state zinc metal battery interface compatibility and delay ion transport kinetics. Cycled anode surface AFM scans showed a smoother PAN‐SiO_2_ membrane‐influenced surface. This is because of the novel way it evaluates; AFM can quantify surface roughness using various features. The vertical distance from the baseline or plane is most often measured. Tannic acid (TA), an organic acid, was used to build a ZnTA anticorrosive coating on the anode.^[^ ^169^ ^]^ The AFM showed a smoother ZnTA anode surface by comparing the height difference between the highest and lowest spots. Peng et al., examined zinc foil surface roughness in various electrolytes for three days and found that trehalose decreased corrosion.^[^ ^170^ ^]^ Our quantitative AFM analysis of surface morphology overcomes initial perceptions to quantify modification effects.

AFM can build a force‐distance curve that precisely captures a materials' Young's modulus by analyzing interatomic attraction and repulsion forces. When evaluating the mechanical stability of AIL, Young's modulus is essential.^[^ ^173^ ^]^ The interfacial layer in this study was TEMPO‐oxidized cellulose nanofiber (TOCNF), a strong electronic insulator. The AFM scanning force curves showed that the TOCNF has four times the mechanical qualities of typical non‐woven paper. Young's modulus mapping was consistent throughout testing. The high modulus homogeneous interfacial layer inhibited dendrite development, allowing Zn||Zn symmetric batteries to cycle for over 300 h at 10 mA cm^−2^. Zhou et al. worked on real‐time characterization and used polyethylene glycol as an additive with Figure 16f showing the lamellar PEG growing bigger as the potential is reducing and gets desorbed as the potential goes to −0.10 V.

#### 4.3.2 Scanning Electron Microscope (SEM)

Electron microscopes use shorter‐wavelength electron beams to achieve nanoscale resolution, while optical microscopes are limited by light wavelength. The SEM uses point‐by‐point scanning to observe superficial features, including deposition morphology and solid electrolyte interfaces at an ultra‐high resolution of 5–10 nm. In alkaline environments, dendrite formation is noticeable and tree‐like, which can puncture the separator and result in a short circuit.^[^ ^174^ ^]^ Flake‐like dendrites in moderate acid environments are less aggressive than tree‐like ones, yet their vertical development can cause battery failure.^[^ ^175^ ^]^ Using SEM, current densities and salt content have been widely studied on dendritic formation.^[^ ^176^ ^]^ The deposition morphology changed from loosely stacked to irregular columnar as the current density grew from 1 to 100 mA cm^−2^. The two loose structures often separate from the anode, forming “dead zinc” across cycles. At 10 mA cm^−2^, the deposition morphology is dense and uniform. The height differences among dendrites decrease because the concentrated electrolyte decreases the ion depletion zone and enhances ion transport because the concentration of salt rises. The SEM images of zinc deposited in the three most common salt systems: ZnSO_4_, Zn(OTF)_2_, and Zn(TFSI) were taken. In these three salt systems, the dendritic morphology was sharply edged with a sheet‐like structure.^[^ ^177^ ^]^ Larger membranes are easier to perforate. The ZIB with Zn(OTF)_2_ has the shortest cycle life, followed by ZnSO_4_, while the high‐concentration salt with 10 m LiTFSI and 1 m Zn(TFSI)_2_ has the longest. Along with dendritic studies, SEM may analyze surface features like AILs. Most AILs are tens to hundreds of nanometres thick, while some organic polymer AILs are microscale, permitting SEM examination. In situ polymerization with dopamine as an electrolyte produces AIL consistency.^[^ ^178^ ^]^ After many cycles, focused ion beam scanning electron microscopy (FIB‐SEM) revealed a polymer layer between the zinc layer and the protective Pt/C. The zinc deposition behavior affected by artificial interphase layers (AILs) has also fascinated researchers. Hao et al. found that zinc ions preferentially accumulated beneath the AIL coating on anodes coated with a highly viscoelastic polyvinylbutyral (PVB).^[^ ^179^ ^]^

#### 4.3.3 In Situ Transmission Electron Microscopy (TEM)

In situ TEM is an exceptionally efficient technique for the atomic‐scale examination of materials. It facilitates the acquisition of localized data on reactive material surfaces, providing insights into the preliminary stages of nucleation. By combining TEM with methods like energy filtration transmission electron microscopy and electron energy‐loss spectroscopy, researchers can examine reacting interfaces and alterations in elemental chemical states with atomic precision. Nonetheless, constraints arise from the ultrahigh‐vacuum prerequisites of TEM and high‐resolution TEM (HR‐TEM), as well as the necessity for materials that can withstand powerful electron beams. The penetrating depth of high‐energy electrons and the requirement for meticulous sample handling present problems. Notwithstanding these factors, TEM/HR‐TEM has been effectively employed in investigations, such as the crystallization of LiFePO_4_ from its amorphous form, elucidating transient crystalline intermediates and phase transitions.^[^ ^180^, ^181^ ^]^ Moreover, Bai et al., worked on aqueous ZIBs and explained the nanosheets' thickness using HR‐TEM.^[^ ^182^ ^]^ Although HR‐TEM is an extremely useful tool, it faces challenges with the compatibility of the electrolytes as rather complex electrolyte chemistries are used to stabilize Zn anode in the lab while only very few simplified electrolyte chemistries are compatible with the TEM setup. Therefore, the response to applied potential in situ TEM could be very different from the actual experimental setup.

### 4.4 Spectroscopic Analysis of Additives in the Electrolytes

Several spectroscopy techniques are used to find the chemical behavior of electrolytes on the Zn surface. This analysis of additives in the electrolyte is essential to understanding the interaction, stability, and impact on electrochemical performance. Techniques such as in situ XRD, SAXS, FTIR, and TOF‐SIMS help to identify the functional group and molecular interaction.

#### 4.4.1 In Situ X‐Ray diffraction Spectroscopy (In‐XRD)

In situ, XRD enables real‐time monitoring of structural evolution in Zn anodes during charge/discharge cycles. This technique allows for the observation of dynamic phase transitions, dissolution/deposition processes, and strain effects in Zn metal.^[^ ^183^ ^]^ By employing an electrochemical in situ XRD setup, researchers can track the reversible formation of Zn‐related intermediates and the impact of electrolyte modifications on structural stability. Therefore, it is crucial to develop strategies for prolonged cycling life and improved Zn anode reversibility. It has been utilized in studies investigating the impact of electrolyte additives such as ZnSO_4_ and Zn(CF_3_SO_3_)_2_ on Zn electrodeposition and passivation. It provides insights into how pH variations and ion interactions influence Zn dendrite growth and suppression mechanisms. This technique is particularly useful in designing protective coatings and ASEI to enhance battery longevity.^[^ ^184^ ^]^ Zeng et al. demonstrated the application of in situ XRD in monitoring the dynamic evolution of Zn metal plating/stripping.^[^ ^185^ ^]^ They observed real‐time phase transitions of Zn anodes under different electrolyte conditions, revealing the reversible formation of ZnO and Zn(OH)_2_ species. Their findings indicated that electrolyte pH plays a significant role in stabilizing Zn anodes by preventing excessive side reactions. Similarly, Zhang et al. employed in situ XRD to investigate Zn deposition behavior on 3D porous Zn scaffolds.^[^ ^186^ ^]^ The results showed that controlled Zn plating led to uniform Zn^2+^ distribution, reducing dendrite formation and enhancing battery longevity. This highlights the critical role of in situ XRD in designing stable Zn anode architectures. Li et al. used synchrotron‐based in situ XRD to analyze Zn‐ion interactions with electrolyte additives and interfacial phase transformations, providing valuable insights into optimizing electrolyte formulations for enhanced cycling stability.^[^ ^187^ ^]^ Despite its advantages, in situ, XRD faces notable challenges in the study of Zn anodes, particularly in detecting crystalline phases at the interface due to their extremely small thicknesses and the poor signal‐to‐noise ratio associated with minor surface phases.

#### 4.4.2 In Situ Raman Analysis

In situ, Raman spectroscopy allows real‐time monitoring of electrolyte composition changes and interfacial reactions occurring at the Zn anode during cycling. This technique is used to analyze dendrite formation, corrosion mechanisms, and SEI layer evolution, providing a better understanding of Zn electrode stability and electrolyte decomposition.^[^ ^188^ ^]^ Several studies have utilized in situ Raman spectroscopy to investigate the stability of ZnSO_4_‐based electrolytes and the role of complexing agents in suppressing unwanted side reactions. Wang et al. demonstrated how Raman spectroscopy was used to track the evolution of sulfate species on Zn anodes in different electrolyte formulations.^[^ ^172^ ^]^ Chen et al. applied in situ Raman spectroscopy to observe SEI layer formation during prolonged cycling, confirming the influence of electrolyte additives in stabilizing the Zn anode.^[^ ^189^ ^]^ In other work, Liu et al. used in situ Raman spectroscopy to detect transient species at the Zn anode‐electrolyte interface, revealing critical insights into SEI formation and growth mechanisms. These findings contribute to optimizing electrolyte formulations to enhance Zn anode performance and longevity. Despite its advantages, Raman spectroscopy in Zn anode studies is limited by fluorescence interference, shallow penetration depth, and challenges in detecting subtle interfacial changes due to laser‐induced artifacts and signal instability.^[^ ^190^ ^]^

#### 4.4.3 Small‐Angle X‐Ray Scattering (SAXS)

**Figure 17:**
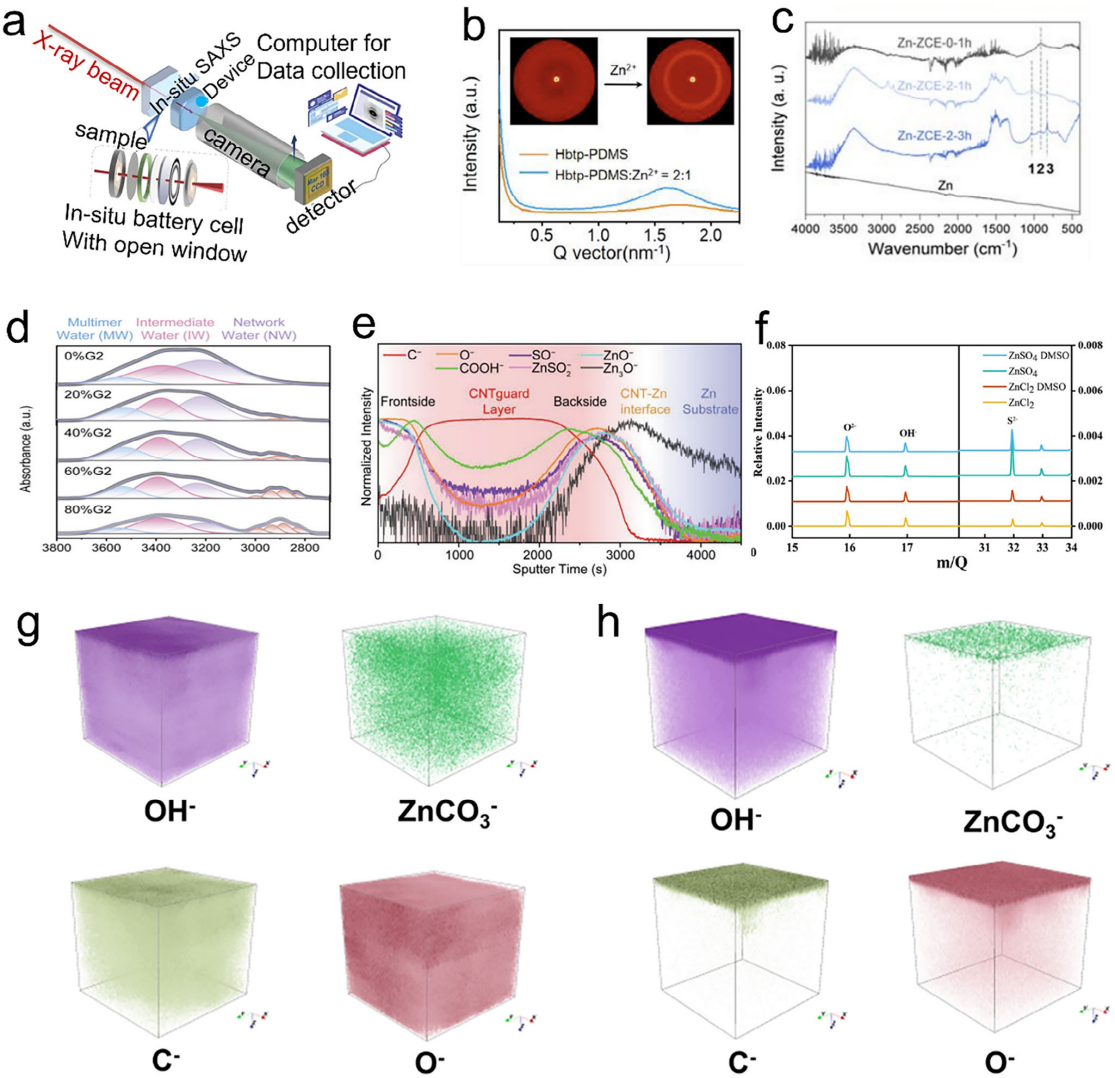
a) SAXS working principal diagram. Reproduced with permission.^[^ ^199^ ^]^ Copyright 2023, Wiley‐VCH. b) SAXS profiles of Hbtp‐PDMS and Zn (Hbtp)₂‐PDMS polymers, with the corresponding 2D SAXS patterns shown in the inset. Reproduced with permission.^[^ ^192^ ^]^ Copyright 2023, Elsevier. FTIR spectra of c) surface layer deposited by different electrolytes. Reproduced with permission.^[^ ^197^ ^]^ Copyright 2023, The Royal Society of Chemistry. d) region from 2700 to 3800 cm^−1^ at room temperature. Reproduced with permission.^[^ ^198^ ^]^ Copyright 2024, The Royal Society of Chemistry. e) Normalized intensity of C^−^, O^−^, COOH^−^, SO^−^, ZnSO_2_^−^ ZnO^−^, and Zn_3_O^−^ in CNTguard‐Zn after cycling along the sputtering time from TOF‐SIMS. Reproduced with permission.^[^ ^200^ ^]^ Copyright 2023, Wiley‐VCH. TOF‐SIMS f) characterization of the O and S elements of the four electrodes. Reproduced with permission.^[^ ^201^ ^]^ Copyright 2023, American Chemical Society. TOF‐SIMS 3D profile g) of BZC/ZCZH/SA@Zn electrode and h) bare Zn electrode after 50 cycles. Reproduced with permission.^[^ ^202^ ^]^ Copyright 2024, Wiley‐VCH.

SAXS is a powerful technique, and the basic principle of SAXS is based on the deflection of X‐rays when they interact with electron‐dense regions within a sample, a process governed by X‐ray scattering. When the sample exhibits a homogeneous electron density, the incident photons scatter predictably, generating a diffraction pattern that is recorded on a 2D detector.^[^ ^191^ ^]^ This data is then converted into a 1D scattering curve related to the azimuthal angle (**Figure** **17**a). SAXS has been recently utilized to investigate the structure evolution of Zn anode during charge/discharge cycling. For example, Fu et al. constructed a supramolecular interface on the Zn surface through the reversibility of dynamic metal‐ligand coordination bonds, which not only suppresses parasitic reactions and corrosion of the Zn anode but also enables high‐rate, high‐capacity Zn anodes. The SAXS profile demonstrates the microstructure of the Zn (Hbtp)_2_‐PDMS polymer (Figure 17b). The increased intensity of the single broad peak of Zn (Hbtp)_2_‐PDMS at 1.62 nm^−1^ represents the coordination microphase separation between the Zn^2+^ and Hbtp complexes. The corresponding distance between the microphases was calculated to be 3.87 nm. This microphase‐separated structure may give the Zn(Hbtp)_2_‐PDMS polymer its high ionic conductivity and mechanical strength.^[^ ^192^ ^]^ SAXS can also be used to test dispersibility. For instance, Yang et al. enhanced the reversibility of Zn anodes by adding hydrophobic carbon dots (CDs) to the ZIB electrolyte to inhibit side reactions with water and promote the desolvation of hydrated Zn ions. The reduced SAXS intensity in the ZnSO_4_ electrolyte‐containing hydrophobic CDs proves good dispersion, which promotes uniform Zinc deposition.^[^ ^193^ ^]^ In addition, SAXS can also be used to observe changes in ion channels. For example, Srinvasan et al. designed a ZIB with a long cycle by using a biomass@Nafion membrane instead of a boat glass fiber membrane to generate SEI on the surface of the Zn anode and changing the coordination of Zn^2+^ in the aqueous medium.^[^ ^194^ ^]^ Lee et al. prepared 3D structured mesoporous carbon (MC) particles with controllable CC bond chemistry and defect structures via carbonized block copolymer (BCP) colloidal particles to improve the cycling and efficiency of zinc anodes and analyzed the key cylindrical morphology of BCP via peak and position analysis in SAXS and determined that the cylindrical morphology was maintained after carbonization.^[^ ^195^, ^196^ ^]^ Despite its merits, SAXS faces challenges such as low spatial resolution, the need for sample homogeneity, the need for ordered/semi‐ordered structures, and interference from the electrolyte.

#### 4.4.4 Fourier Transform Infrared Spectroscopy (FTIR)

In ZIBs, it is crucial to determine the performance of water because of aqueous electrolytes. As polar molecules are susceptible to water, FTIR is a reasonable technique for water analysis. The transition among molecular vibrational energy levels reveals chemical bonding and functional groups applicable for electrolyte decomposition analysis. For the O‐H stretching vibration of water, the band ranges from 2800–3700 cm^−1^ and several coordination states of water are determined via peak fitting analysis.^[^ ^190^ ^]^ FTIR spectra show the surface layer deposition through different electrolytes and reveal the peaks related to C‐N and C‐H bonds. It also affirms the increasing trends of vibrational peaks of C‐O and Zn^2+^ complex ascending at peaks 1 and 3, whereas OH vibration at peak 2 for Zn_5_(OH)_8_Cl_2_.H_2_O (Figure 17c).^[^ ^197^ ^]^ Moreover, organic molecules can directly integrate to assist anions in altering the solvation structure. Yang et al. used sodium dodecyl benzene sulfonate (SDBS) as an additive in the electrolyte and FTIR spectrum, suggesting the presence of SDBS on the Zn surface.^[^ ^137^ ^]^ C‐H vibrations of organic SDBS prompt peak spectrum at 2850, 2928, and 2956 cm^−1^. Whereas the 1450 cm^−1^ peak shows the presence of the phenyl group in SDBS. Liu et al. selected a dual‐functional organic electrolyte additive tripropyleneglycol (TG), which regulates the solvation structure and Zn deposition.^[^ ^133^ ^]^ Reversible conversions in hydroxyl groups represent the reversible adsorption and desorption manner of solvated water studied through FTIR images, which reveals that TG can reversibly influence hydrogen bond groups. Generally, functional additives are used to adjust the solvation structure and control the deposition process, while the residues can adjust the pH value of the electrolyte. Mostly, the solvation of additives is estimated by polarity, which is further evaluated by the electric constant and dipole moment. Molecules with large polarity compared with water molecules show strong bonding energy with Zn‐ions and prevent proton hopping among water molecules, ultimately enhancing the energy obstacle for H_2_‐bond collapse and reconstruct. Zhang et al. introduced a co‐solvent diethylene glycol dimethyl ether (G2) in weakly solvating aqueous electrolytes for extreme temperature (−60 °C to 60 °C) operations in ZIBs.^[^ ^198^ ^]^ It was discovered by using in situ FTIR that G2 molecules bind with reactive desolvated water and inhibit the water contact with the electrode surface, which ultimately prevents side reactions for above 7500 h (Figure 17d).

#### 4.4.5 Time‐of‐Flight Secondary Ion Mass Spectrometry (TOF‐SIMS)

ToF‐SIMS is a surface‐sensitive analytical technique that provides detailed elemental, isotopic, and molecular information about surfaces, interfaces, and thin layers with nanoscale spatial resolution and parts per billion sensitivities, which are advantageous for detecting trace impurities, corrosion products, and nucleation sites for dendrite formation on zinc anodes. To analyze the stripping and plating phenomenon in ZIBs, TOF‐SIMS in anode ion mode is used to see the material distribution on Zn after the cyclic deposition and to characterize the composition of solid surfaces at nanometre‐scale resolution. This technique yields comprehensive chemical and molecular details by revealing secondary ions emitted from the material's surface due to the bombardment of high‐energy ion beams.^[^ ^203^ ^]^ Zhou et al. introduced charge carriers during the Zn plating by applying zincophilic carbon nanotubes (CNTs) on the Zn anode (Figure 17e).^[^ ^200^ ^]^ TOF‐SIMS was used to investigate the mechanism of plating and striping, and numerous COOH^−^ were detected at the surface CNT layer, where the electrochemical hydrophilic reaction occurred. As sputtering time increased, COOH‐ and C‐ intensity reduced. Zn_3_O^−^ signals the Zn with less oxidation and shows the Zn metal below the CNT guard layer. Hu et al. worked on SEI formation on zinc metal anode to avoid dendrites, HER, and other side reactions.^[^ ^201^ ^]^ TOF‐SIMS identified three main components on the SEI surface: S^2−^, OH^−^, and O^2−^. Furthermore, S‐based components were more due to the presence of ZnSO_4_ electrolytes in comparison to the ZnCl_2_‐based electrolytes. Whereas, by adding DMSO in ZnSO_4_, O and S peaks decrease and prove that by the addition of DMSO, the amount of S^2−^ and OH^−^ decreases (Figure 17f). Zhou et al. introduced a solid‐to‐hydrogel electrolyte interface for uniform deposition on the Zn anode.^[^ ^202^ ^]^ 3D profile of TOF‐SIMS identified the evenly distributed C, O, and OH (Figure 17g,h). Further, ZnCO_3_^−^ at negative mode exhibits after 50 cycles, and in the bare Zn electrode, ZnCO_3_^−^ is not detectable after cycling. More specifically, the TOF‐SIMS enables high‐resolution chemical mapping of surface layers and by‐products such as Zn(OH)₂, ZnO, and sulfate salts, helping identify dendrite precursors and interfacial degradation on zinc metal anodes. Guifang Zeng et al.,^[^ ^204^ ^]^ investigated the influence mechanism of Zn(CF_3_COO)_2_ electrolyte additives on the in situ formation of inorganic SEI using TOF‐SIMS. Based on the ToF‐SIMS results show that in the presence of byproduct ZSH, the S element is generated during cycling in the ZS electrolyte. Although TOF‐SIMS has shown great promise in revealing surface chemistry of the zinc anodes, however the technique suffers from shallow probing depth and the potential beam‐induced damage to the zinc anode.

#### 4.4.6 X‐Ray Computed Tomography (XCT)

X‐ray Computed Tomography (XCT) is a powerful non‐destructive imaging tool for visualizing and quantifying internal microstructural changes in Zn anodes during battery operation. With high spatial resolution and 3D capability, XCT enables insights into failure mechanisms such as dendrite growth, void formation, and structural degradation across cycling. Yufit et al. used high‐resolution synchrotron XCT to provide the first real‐time 3D visualization of zinc dendrite formation, growth, and dissolution, revealing persistent porous structures and enabling correlation between morphological evolution and electrochemical performance in Zn anodes.^[^ ^39^ ^]^ As summarized in Figure 3b, the 3D XCT provided evidence about dendrite growth indicating the impact of voltage, local current density and temperature on nucleation and initiation of dendrites. Similarly, Chen et al., studied the real‐time 3D visualization of zinc morphology evolution and gas void formation during cycling, providing critical insights into degradation mechanisms in zinc‐air batteries by using XCT.^[^ ^205^ ^]^ In essence, the 3D XCT has evolved as a great tool to analyze the electrode morphology and visualize the dendrite growth, however, the technique faces several challenges including sample deformation and possibility to use limited current densities only etc.

In summary, the modern characterization techniques have significantly advanced the understanding of Zn anode behavior by enabling real‐time observation of interfacial processes and structural evolution under operating conditions. Methods such as in situ spectroscopy, advanced electron microscopy, and synchrotron‐based imaging provide critical insights into dendrite formation, hydrogen evolution, and surface instability. These tools are critical for uncovering degradation pathways and guiding the rational design of more durable and efficient Zn‐based batteries.

## 5 Conclusion and Future Prospects

ZIBs are promising (ESS) due to their inherent advantages of low cost, high safety, and environmental friendliness. On the contrary, substantial challenges remain, particularly with the zinc anode, which experiences dendritic formation, corrosion, passivation, and HER. These concerns destructively impact the long‐term stability, efficiency, and performance of ZIBs. This comprehensive review has broadly summarized the key challenges zinc anode faces and proposed various stabilization strategies focusing on structural and interface engineering to alleviate these issues. By incorporating 3D structural designs, homogeneous mass transfer can be achieved, which is crucial in reducing dendrite formation and ensuring even ion distribution during charge/discharge cycles. Additionally, nanostructuring of alloy or composite material‐based anodes has been explored as an effective approach to enhance mechanical stability, mitigate volume changes, and improve overall electrochemical performance. Moreover, we discussed advanced interface engineering techniques, such as regulating the solvation structure to stabilize the electrolyte, forming adsorption layers via artificial SEI and in situ SEI that can protect the Zn anode, and forming electrostatic shielding effects to prevent dendritic growth. These strategies show considerable promise in suppressing unwanted side reactions and improving the reversibility of zinc plating/stripping processes to enhance the overall performance of ZIBs.

### 5.1 Future Prospects

Future research on ZIBs must address the persistent challenges associated with the zinc metal anode, particularly in enhancing electrochemical performance, long‐term stability, and operating safety. Here, we summarize a few of the emerging directions which hold considerable promise.

#### 5.1.1 Advanced Interface Engineering and Electrolyte Design

Stabilizing the Zn anode–electrolyte interface remains a priority for achieving reliable ZIBs. Future strategies should focus on constructing protective interphases, optimizing Zn^2+^ transport, and suppressing parasitic reactions such as hydrogen evolution. The use of hydrogel‐based electrolytes has shown notable success. For instance, Wang et al. developed a sodium alginate hydrogel modified with a PEDOT:PSS polymer layer to enable dual ion–electron transmission channels, effectively mitigating dendrite growth and side reactions, leading to over 6700 h of stable cycling.^[^ ^206^ ^]^ This approach demonstrates the promise of multifunctional electrolyte frameworks in promoting uniform Zn plating and long‐term stability. Similarly, Li et al. employed a pyridine oxide additive to reconstruct the Helmholtz plane, achieving compact Zn^2+^ deposition and suppressing active water‐induced parasitic reactions.^[^ ^207^ ^]^ This approach resulted in stable performance exceeding 3000 cycles even under high mass loading, and represents a refined control over interfacial chemistry at the molecular scale.

#### 5.1.2 Temperature‐Resilient Electrolyte Systems

Conventional aqueous electrolytes suffer from limited temperature adaptability. Expanding the operational window is essential for real‐world ZIB deployment. Xu et al. introduced a covalent organic framework (COF)‐based separator with quasi‐single‐ion Zn^2+^ conductivity.^[^ ^208^ ^]^ Their COF‐Zn membrane, coupled with a tailored electrolyte, supported efficient operation from −40 to 100 °C while minimizing cathode dissolution and improving Zn anode reversibility. Similarly, Chen et al. proposed a hydrated deep eutectic solvent system that formed a zincophilic gradient SEI layer, enabling stable performance from −30 to 60 °C.^[^ ^209^ ^]^ Such designs can facilitate broader electrochemical windows, suppress hydrogen evolution, and enhance interfacial stability across diverse operating conditions. These advances highlight the need for tailored ion transport media that enable consistent battery performance under varying climatic and operational conditions.

#### 5.1.3 Understanding and Modulating Zn^2+^ Solvation and Hydrogen Bond Networks

A deeper understanding of solvation chemistry is crucial for designing next‐generation electrolytes. Yang et al. demonstrated that a chitosan oligosaccharide (COS) layer could construct a hydrogen‐bonding network on the Zn surface, enhancing desolvation kinetics and Zn^2+^ transport.^[^ ^210^ ^]^ This modification led to significant improvements in Coulombic efficiency and cycle life. Exploring such bio‐inspired and molecularly interactive interfaces could yield new design rules for electrolyte engineering.

#### 5.1.4 Rational Structural Engineering of Zinc Anodes

Surface and bulk structural modifications play an important role in regulating local current densities and Zn deposition pathways. Zhang et al. applied picosecond laser lithography to create a micro‐nano surface structure that reduced electrolyte contact, enhanced corrosion resistance, and directed Zn^2+^ deposition along the (002) plane, achieving high Coulombic efficiency and long‐term cycling stability.^[^ ^211^ ^]^ These engineered textures represent a scalable route to suppress dendritic growth through surface energy modulation. Zhang et al. took a different approach by introducing amino acid‐based additives to engineer Zn(002) planes, promoting layered 3D structures and suppressing dendrite growth.^[^ ^212^ ^]^ The resulting anodes delivered stable cycling under high current conditions and substantial capacity retention. Such additive‐induced crystallographic control offers an attractive pathway to more reversible Zn plating and stripping.

#### 5.1.5 Real‐Time Understanding through Advanced Characterization Tools

The application of in situ and operando techniques will be central to future breakthroughs. Techniques such as synchrotron‐based XRD, in situ TEM, and operando spectroscopy allow real‐time monitoring of dendrite growth, SEI formation, and interfacial evolution. Zhang et al. reviewed recent developments in situ characterization for Zn anodes, highlighting their role in identifying failure mechanisms and guiding rational design.^[^ ^213^ ^]^ Integration of these methods into iterative experimental workflows will be critical to demystifying complex electrochemical behaviors.

#### 5.1.6 Data‐Driven Material Discovery and AI Integration

Artificial intelligence and machine learning are emerging as powerful tools for accelerating battery development. Zakerabbasi et al. demonstrated that Gaussian Process Regression models could predict metal battery energy density with near‐perfect accuracy, revealing key material‐property relationships.^[^ ^214^ ^]^ These methods streamline the design process and reduce reliance on trial‐and‐error experimentation.

In a related development, Zhu et al. used machine learning to select polyacrylamide‐based gel polymer electrolytes, achieving high mechanical and electrochemical performance while suppressing dendrite growth.^[^ ^215^ ^]^ Similarly, Luo et al. developed an ML‐assisted model to predict Gutmann donor numbers of organic additives, enabling rapid electrolyte additive screening and experimental validation.^[^ ^216^ ^]^ As data collection improves, these approaches will likely play an increasingly central role in guiding material discovery in ZIB research.

As ZIBs continue to evolve, integrating insights from advanced characterization, interface science, and data‐driven design will be vital for overcoming longstanding performance barriers. The convergence of these multidisciplinary approaches offers a promising pathway toward building robust, scalable, and sustainable zinc‐based energy storage systems. With continued innovation, ZIBs are poised to play a pivotal role in the future of grid‐scale and decentralized energy applications.

## Conflict of Interest

The authors declare no conflict of interest.
